# Multiscale influenza forecasting

**DOI:** 10.1038/s41467-021-23234-5

**Published:** 2021-05-20

**Authors:** Dave Osthus, Kelly R. Moran

**Affiliations:** 1 Los Alamos National Laboratory, Statistical Sciences Group, Los Alamos, NM USA; 2 Department of Statistical Science, Duke University, Durham, NC USA

**Keywords:** Influenza virus, Epidemiology, Statistics

## Abstract

Influenza forecasting in the United States (US) is complex and challenging due to spatial and temporal variability, nested geographic scales of interest, and heterogeneous surveillance participation. Here we present Dante, a multiscale influenza forecasting model that learns rather than prescribes spatial, temporal, and surveillance data structure and generates coherent forecasts across state, regional, and national scales. We retrospectively compare Dante’s short-term and seasonal forecasts for previous flu seasons to the Dynamic Bayesian Model (DBM), a leading competitor. Dante outperformed DBM for nearly all spatial units, flu seasons, geographic scales, and forecasting targets. Dante’s sharper and more accurate forecasts also suggest greater public health utility. Dante placed 1st in the Centers for Disease Control and Prevention’s prospective 2018/19 FluSight challenge in both the national and regional competition and the state competition. The methodology underpinning Dante can be used in other seasonal disease forecasting contexts having nested geographic scales of interest.

## Abstract (web summary)

Influenza forecasting in the United States is challenging and consequential, with the ability to improve the public health response. Here the authors show the performance of the multiscale flu forecasting model, Dante, that won the CDC’s 2018/19 national, regional and state flu forecasting challenges.

## Introduction

Influenza represents a significant burden to public health with an estimated 9 to 49 million cases each year in the United States (US)^1^. Influenza (flu) related activity is monitored in the US by the Centers for Disease Control and Prevention (CDC) through numerous surveillance efforts. One such effort is the Outpatient Influenza-like Illness Surveillance Network (ILINet). ILINet collects weekly data on influenza-like illness (ILI) from over 2000 healthcare providers from all 50 states, Puerto Rico, the US Virgin Islands, and the District of Columbia. ILI is defined as a temperature greater or equal to 100 °F, a cough or sore throat, and no other known cause, representing symptoms consistent with influenza. ILINet constitutes a significant and necessary effort to understanding the spread and prevalence of flu-like illness in the US in near real-time.

With mature ILI surveillance infrastructure in place in the US, attention has turned in recent years to ILI prediction. The ability to predict the spread of ILI poses a substantial public health opportunity if able to be done accurately, confidently, and with actionable lead times at geographic and temporal scales amenable to public health responsiveness. Since 2013, the CDC has hosted an influenza forecasting challenge called the FluSight challenge to gauge the feasibility of forecasting targets of public health interest in real-time, to galvanize the flu forecasting community around common goals, and to foster innovation and improvement through collaboration and competition^2–4^. The FluSight challenge has been a leading driver of recent model development and flu forecasting advancements^5–21^.

Up until the 2016 flu season (i.e., the flu season starting in the fall of 2016 and ending in the spring of 2017), the FluSight challenge’s scope encompassed probabilistic forecasting of short-term (1 to 4 week ahead) and seasonal (season onset, peak timing, and peak intensity) targets at two geographic scales: nationally and regionally, where regions correspond to Health and Human Services (HHS) regions. Probabilistic forecasting is carried out by binning the support of the targets (e.g., binning the peak timing target into weeks of the season) and assigning a probability to each bin, representing the probability the eventual outcome will fall in each bin. Probabilistic forecasts are a crucially important component of the FluSight challenge, as it demands not only information on what the forecasting models thinks will happen, but also how confident the forecasting model is in its own prediction. National and regional forecasts give a high-level view of flu activity across the US. Those forecasts provide value to national and regional public health officials, but offer only coarse information for state and local public health practitioners. Thus, starting with the 2017 flu season, the FluSight challenge expanded to a third geographic scale: states and territories (referred to as states). This expansion to a finer geographic scale presents an opportunity to move forecasting to geographic scales better aligned with public health response infrastructure and decision making. It also presents an opportunity to develop and advance methodological forecasting frameworks that can share information across geographic locations, flu seasons, and geographic scales coherently in ways that geographically isolated forecasting models cannot.

**Fig. 1:**
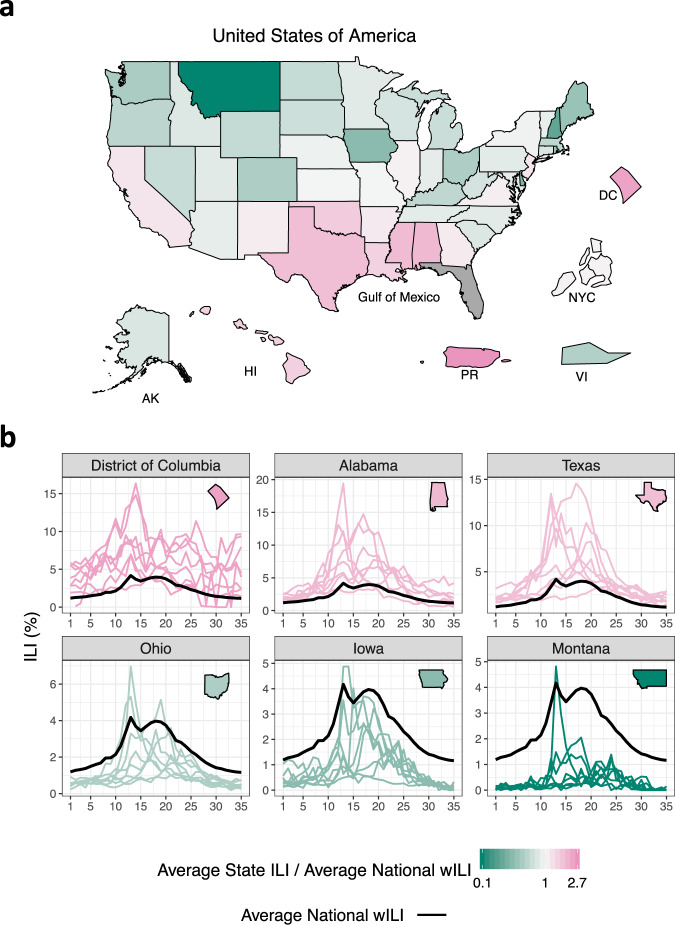
State-to-state influenza-like illness variability. **a** Average state influenza-like illness (ILI) relative to average national weighted ILI (wILI). States bordering the Gulf of Mexico tend to have higher ILI than the national average. The geographical sizes of Alaska (AK), Hawaii (HI), Puerto Rico (PR), the US Virgin Islands (VI), New York City (NYC), and the District of Columbia (DC) are not to scale. Data for Florida is unavailable. Averages are based on 2010 through 2017 data. **b** ILI by season (colored lines) for select states. Black line is national average wILI for reference. Appreciable season-to-season and state-to-state ILI variability exists.

Multiscale forecasting in the US requires careful consideration as it presents numerous challenges. For instance, Fig. 1 (as well as Supplementary Fig. 3) shows appreciable state-to-state ILI variability. As an example, Montana’s average ILI is about 20% the national average, while the District of Columbia’s and Puerto Rico’s average ILI is about 250% the national average. Figure 1 also shows evidence of spatial correlation, with states near the Gulf of Mexico having higher than average ILI while most Midwest and Mountain West states have lower than average ILI. Attempts to model the spatial relationships of flu and flu-like illnesses include using network models and US commuter data^5^, network models based on Euclidean distance^19^, and empirically derived network relationships^21,22^. Though these approaches consider spatial relationships differently, they all support the conclusion that sharing information across geographical units can improve forecasting.

**Fig. 2:**
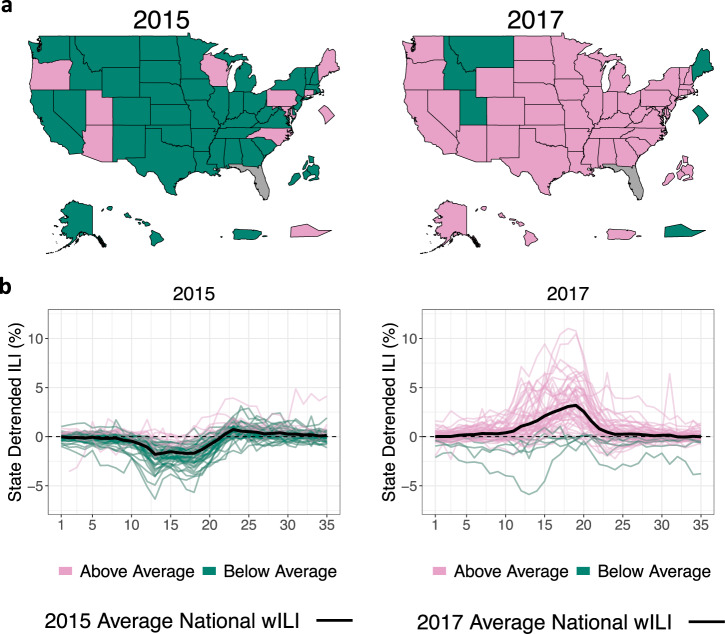
Seasonal influenza-like illness variability. **a** Dark green states denote states with ILI less than their state-specific averages while pink states are states with ILI above their state-specific averages. 2015 was a mild flu season for the majority of states relative to their state-specific average ILI, while 2017 was an intense flu season for the majority of states, indicating that season-to-season effects can affect most of the country. Data unavailable for Florida. States displayed outside of the contiguous US are geographically not to scale. **b** State detrended ILI for the 2015 and 2017 flu seasons, where state detrended ILI is ILI for a state/season minus ILI for that state averaged over all seasons. Positive/negative state detrended ILI means ILI for that season was above/below the state-specific average, respectively. Black line is season-specific national average wILI for reference.

Figure 2 (and Supplementary Fig. 4) shows season-to-season variability, illustrating the common directional effect a flu season can have on nearly all states. 2015, for instance, was a mild flu season in the US with 42 out of 53 states experiencing ILI activity below their state-specific averages (the 53 states are all 50 states, minus Florida with no available data, plus Puerto Rico, the US Virgin Islands, New York City, and the District of Columbia). In contrast, 2017 was an intense flu season with 47 out of 53 states experiencing ILI activity above their state-specific averages. Supplementary Fig. 5 visualizes this information with six intensity levels rather than an above/below average binary. Similar to the findings that sharing information across geographical units can improve flu forecasting, previous work has found that sharing information across seasons can also improve flu forecasting^13^. Similarly positive findings have been identified in other disease modeling contexts using latent random walks^23,24^.

**Fig. 3:**
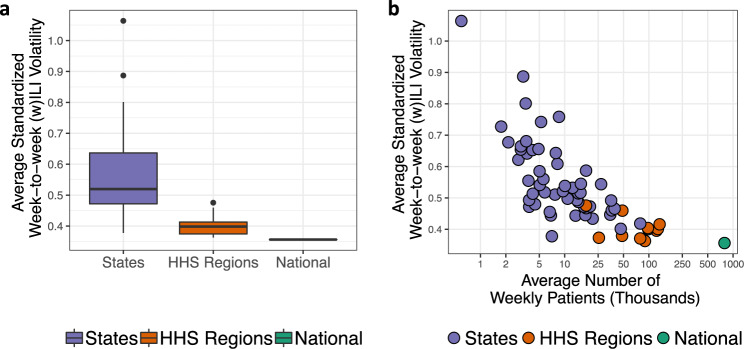
Standardized volatility by geographic scale. **a** Average standardized week-to-week influenza-like illness (ILI—states) and weighted ILI (wILI—HHS regions and nationally) volatility for three geographic scales. Volatility decreases as the scales coarsen. Boxplots present median (center line), interquartile range (boxes), 1.5 times the interquartile range (whiskers), and outliers (points) based on *n* = 53, 10, and 1 observations for states, HHS regions, and the nation respectively. **b** Average standardized week-to-week (w)ILI volatility versus the average number of weekly patients on a log scale for each state, HHS region, and nationally. Volatility decreases as the number of weekly patients seen increases, suggesting that volatility is in part a product of ILINet participation.

Figure 3 shows the average standardized week-to-week volatility across geographic scales (see Supplementary Note 3 for details). Standardized volatility measures how much ILI (states) and wILI (regions and nationally) varies from week-to-week, where wILI is weighted ILI—a state-population weighted version of ILI used to characterize ILI regionally and nationally. High volatility poses a challenge to forecasting as increased volatility can swamp the signal in the (w)ILI data. Figure 3 makes clear that extending forecasts down to the state scale, a more actionable scale for public health officials, comes at the cost of increased volatility. The level of volatility is largely driven by the number of patients seen weekly, as illustrated in Fig. 3. Developing multiscale flu forecasting models that account for decreasing volatility with coarsening geographic scales will be crucial. Some multiscale forecasting models have been developed in the context of norovirus gastroenteritis prediction^25^. In that work^25^, showed that modeling at the finest available data scale and aggregating up to coarser scales generally had better predictive performance than models directly operating at the aggregated scales. To our knowledge, such models have not been developed and operationalized for ILI forecasting.

It is in the context of appreciable state-to-state and season-to-season variability, uneven ILINet surveillance participation, and the need to render short-term and seasonal probabilistic forecasts at nested geographic scales that Dante, a probabilistic, multiscale flu forecasting model, was developed. While efforts have been made to address each of these challenges in isolation, no one has yet to tackle all of these challenges simultaneously in the context of influenza forecasting. Jointly addressing all these challenges is the main contribution of this paper.

Dante is composed of two submodels: a fine-scale model for the state scale and an aggregation model for the regional and national scales. The state submodel includes both a data model and a process model. The data model is where Dante learns information about the level of volatility in the ILI time series. The process model is where Dante captures common and specific structure in the data, including a term common to all states and seasons, a state-specific term, a season-specific term, and a state-season specific interaction term. The common term can be thought of as the average profile of the ILI data across space and time, whereas the interaction term captures deviations from common, state-, and season-specific baselines. The aggregation model builds regional and national forecasts using the state forecasts as population weighted building blocks, leading to forecasts that are coherent across geographic scales. Full details of both submodels are provided in the Methods section.

## Results

Dante is compared to a leading flu forecasting model, the Dynamic Bayesian Model (DBM)^13^. Like Dante, DBM models ILI data as the sum of component trajectories. DBM’s components include a season/region-specific susceptible-infectious-recovered (SIR) compartmental model, a region-specific statistical discrepancy component capturing deviations from the SIR component common across seasons (e.g., holiday effects), and a regularized season/region-specific discrepancy component to capture ILI structure unable to be captured by the other two components. DBM is fit to each geographic unit separately, thus does not share information across geographic units or geographic scales, but does share information across flu seasons. In contrast, Dante shares information across geographic units, geographic scales, and flu seasons. DBM was the fourth place model and a component model in the second place ensemble model^18^ in the prospective national and regional 2017/18 FluSight challenge out of 29 participating models. Dante was the first place model in the 2018/19 national and regional FluSight challenge out of 33 participating models. Dante also came in first out of 14 competing models in the 2018/19 state challenge.

We compare Dante and DBM using forecast skill following the scoring rules of the CDC’s FluSight challenge (details in Supplementary Note 8), noting both that this scoring rule is improper^18,26,27^ and that a proper scoring rule has been implemented in the FluSight challenge starting with the 2019/20 season. In this paper, forecast skill is defined as the exponentiated average over forecast scores and ranges between 0 and 1, with 1 being the best possible forecast skill. Put another way, skill here is the geometric average probability assigned to the observed outcome or, in the case of the CDC’s multibin score, values reasonably close to the observed outcome. This differs from the definition of skill in^28^ defining an average over forecast scores in relation to a reference forecast. Conceptually, skill is a function of both accuracy (a measure of a point summary of a distributional forecast) and sharpness (a measure of concentration of the distributional forecast). We also score both models using a proper log scoring rule (details in Supplementary Note 8.3). We will show how Dante compares to DBM broadly in terms of skill, and also in terms of its component pieces. Both models were fit in a leave-one-season out fashion, where the data for all seasons not being forecasted along with the data for the season being forecasted up to the forecast date were used for training.

Table 1 shows that Dante outperformed DBM in forecast skill at all geographic scales as calculated by both the improper CDC scoring rule and a proper log scoring rule. Dante also outperformed DBM in terms of accuracy, as measured by mean squared error (MSE) of point predictions (posterior means), at all geographic scales. For both models, forecast skill improves and average MSE decreases as geographic scales coarsen, suggesting that both forecast skill and accuracy degrade as we move to finer scales where volatility is greater. See Supplementary Note 12 for further details and figures comparing the MSE of Dante and DBM.

**Table 1:** Comparison of forecast performance between Dante and DBM.

Metric	Model	States	HHS Regions	National
Skill	Dante	0.372 (0.060)	0.413 (0.073)	0.439 (0.078)
	DBM	0.337 (0.053)	0.383 (0.066)	0.426 (0.074)
MSE	Dante	3.164	2.441	1.921
	DBM	3.390	2.674	2.244

Dante and DBM average forecast skill and mean squared error (MSE) comparisons across geographic scales. For skill results both the FluSight challenge’s forecast skill and the exponentiated, proper log score are reported, with the proper skill in parentheses. Forecast skill and MSE each improve for both models as the geographic scale coarsens. Dante outperformed DBM at all geographic scales under both performance metrics.

**Fig. 4:**
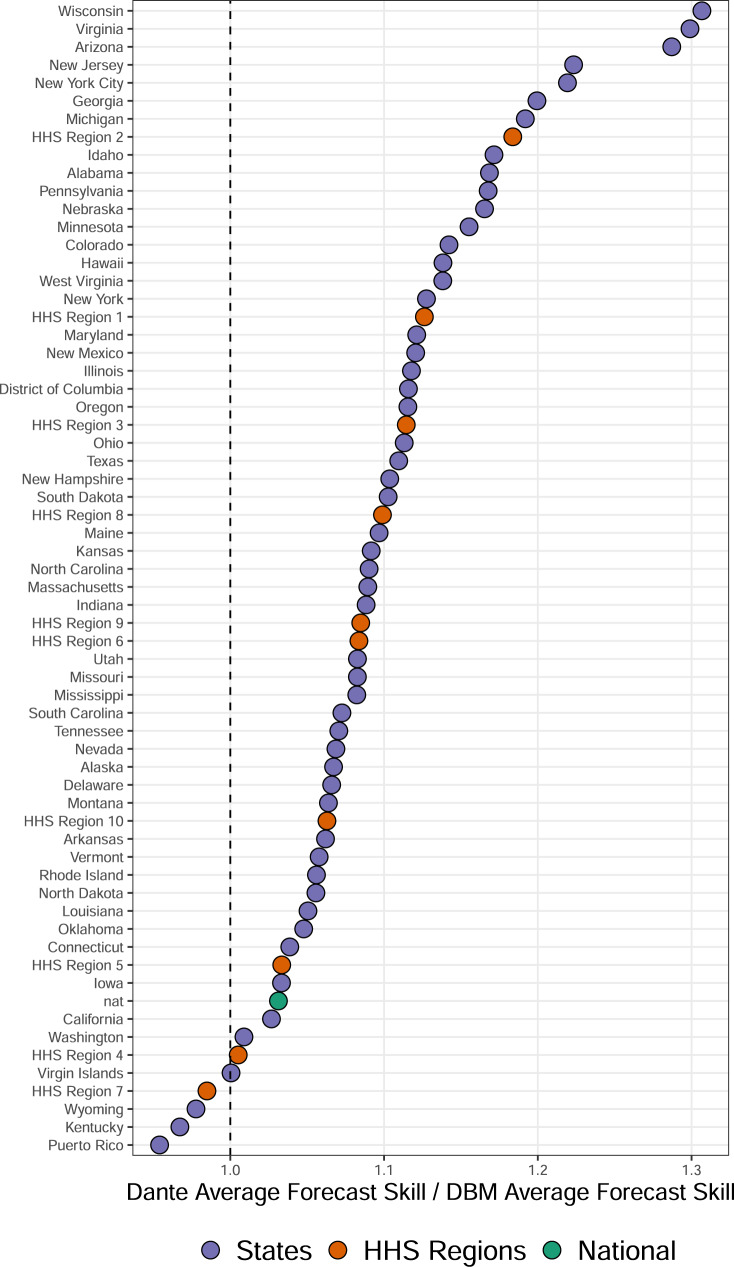
Dante’s forecast skill relative to DBM’s by geographic region. Ratio of forecast skill of Dante to that of DBM, for all states, regions, and nationally. Dante had higher forecast skill for all geographic regions except for HHS Region 7, Kentucky, Wyoming, and Puerto Rico.

Figure 4 shows the ratio of forecast skill of Dante to that of DBM for each state, region, and nationally. Dante outperformed DBM for the majority of geographic regions, with the exception of HHS Region 7 and the states Wyoming, Puerto Rico, and Kentucky.

**Fig. 5:**
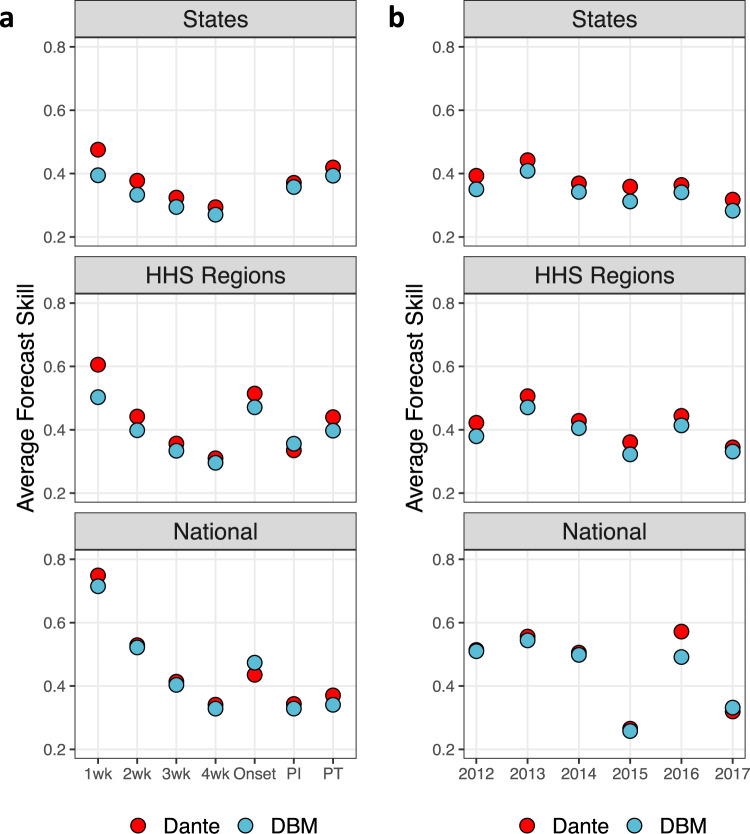
Dante’s and DBM’s forecast skill by target and season. **a** Average forecast skill by scales and targets. PI and PT stand for peak intensity and peak timing, respectively. Dante outperformed DBM for all scales and targets, except for onset nationally and for PI regionally. **b** Average forecast skill by scales and flu seasons. Dante outperformed DBM for all scales and targets, except for 2017 nationally.

Figure 5 shows forecast skill broken down by targets (left) and flu seasons (right) for each geographic scale. Dante outperformed DBM for all scales and targets, except for peak intensity regionally and onset nationally. Improvement over DBM is largest for the 1-week ahead forecast target. For context, in the 2018/19 FluSight national and regional challenge, Dante placed first for all short-term targets, season onset, and peak intensity (PI), while placing ninth for peak timing (PT). In the state challenge, Dante placed first in all short-term targets and PT, while placing second for PI. Dante also outperformed DBM for all scales and flu seasons, except for 2017 nationally. While forecast skill for DBM and Dante are close for all seasons nationally (sans 2016), Dante consistently and appreciably outperformed DBM for all seasons at the regional and state scales.

**Fig. 6:**
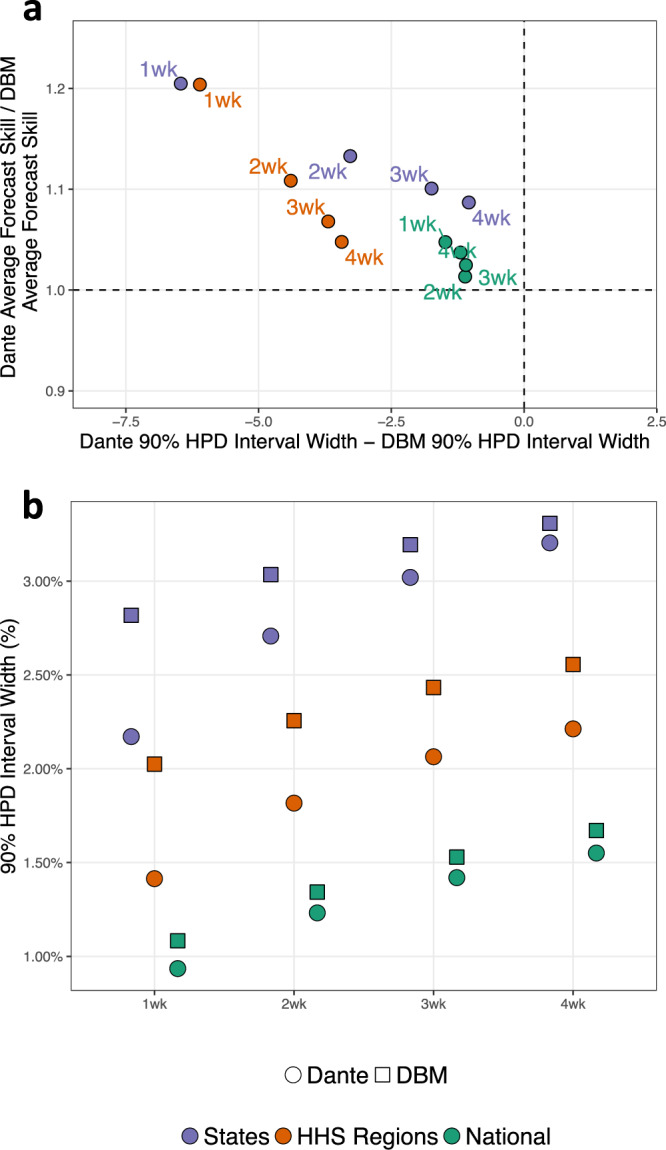
Dante’s and DBM’s short-term forecast skill and sharpness by geographic scale. **a** Ratio of average forecast skills versus difference in 90% highest posterior density (HPD) interval widths for short-term forecasting targets. For all short-term forecasting targets and geographic scales, Dante produced sharper (i.e., smaller 90% HPD interval widths) and higher scoring forecasts than DBM. **b** Average 90% highest posterior density (HPD) interval widths for the short-term forecasting targets. Both Dante and DBM produce sharper (i.e., smaller 90% HPD interval widths) forecasts for coarser geographic resolutions. For all short-term forecasting targets and geographic scales, Dante produced sharper forecasts than DBM.

Figure 6a provides context as to how Dante is outperforming DBM. Figure 6a displays the ratio of forecast skills at each scale for all short-term forecasts against the difference in the 90% highest posterior density (HPD) predictive interval widths for each of the short-term target’s posterior predictive distributions. See Supplementary Note 9 for calculation details. HPD predictive intervals are similar to equal-tailed predictive intervals as they capture the range of probability concentration, but are more appropriate than equal-tailed predictive intervals for distributions that are not unimodal and symmetric. Figure 6a shows that for all scales and short-term forecasts, Dante has smaller HPD interval widths, indicating that Dante’s forecasts are more concentrated (ı.e., sharper) than DBM. Dante’s increased forecast sharpness resulted in higher forecast skill than DBM. This is a promising finding, as sharper forecasts, if well-calibrated, provide more information to public health decision makers.

Figure 6b shows that Dante’s forecasts are sharper than DBM’s for all short-term targets at all geographic scales. For each short-term target, forecasts for both DBM and Dante become sharper as the geographic scale coarsens. DBM makes sharper short-term forecasts because the (w)ILI DBM is modeling less volatile, i.e., because (w)ILI becomes less volatile as geographic scales coarsen. Dante makes sharper short-term forecasts at coarsening geographic scales as a result of the aggregation model. Dante’s 3-week-ahead 90% HPD interval widths nationally and regionally are 1.4 and 2.1%, respectively, about the same as Dante’s 1-week-ahead 90% HPD interval widths are regionally and at the state-level, respectively. Said another way, Dante loses about 2 weeks of sharpness in its short-term forecasts for each disaggregating geographic scale.

**Fig. 7:**
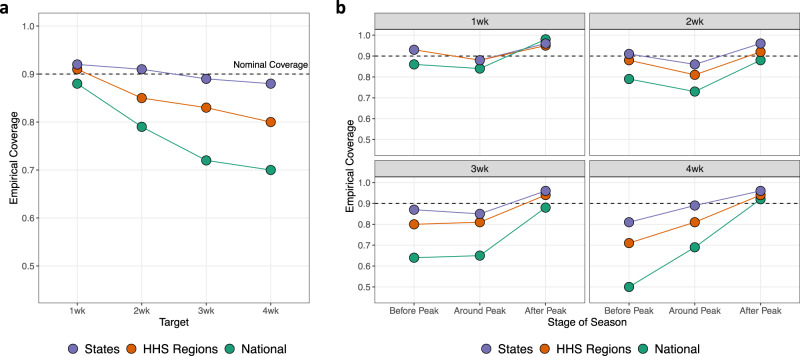
Dante’s empirical coverage. Dante’s 90% empirical coverages for short-term targets, broken down by geographic scales. **b** Empirical coverages by target averaged over all seasons, geographic units within scale, and stages of flu season. **a** Empirical coverages broken out by stages of flu season. The “Around Peak” stage is defined as the peak week, plus/minus 2 weeks inclusively. Generally, empirical coverages degrade as the forecast window increases, as geographic scales coarsen, and as we get earlier in the flu season. The disagreement between empirical and nominal coverage for Dante represents an opportunity for iteration and improvement.

Though Dante won the 2018/19 FluSight challenge and outperformed a leading flu forecasting model, DBM, in a retrospective comparison, Dante is still a work in progress. Figure 7 shows Dante’s 90% empirical coverages for short-term targets and represents an opportunity for future Dante improvement. Overall, empirical coverages for states are close to their nominal coverages. Those empirical coverages drop, however, as geographic scales coarsen. Furthermore, empirical coverages drop with a growing forecast window. The right of Fig. 7 breaks down empirical coverages into the stages relative to the season peak. Empirical coverages are generally good in the post-peak stage of the flu season, arguably when forecasts are least useful. Fig. 7 makes clear that while Dante represents the state of the art, flu forecasting is a field ripe for improvement, advancement, and innovation.

## Discussion

The plot of skill score (Fig. 5) and total patients seen and volatility (Fig. 3) suggests that Dante (and DBM) can forecast geographic regions better when the forecasted estimate is based on more data than less. This result suggests that expanded ILI surveillance participation plays a role in improving model forecasts, not just improvements to the models themselves. This idea is not surprising. Disease forecasting has been compared to weather forecasting^29^, a field that has continued to make consistent progress through parallel efforts of improved modeling and data collection.

We found that Dante made sharper forecasts, as measured by smaller 90% HPD interval widths than DBM, a model that fits aggregate data directly. Similar findings were noted by^25^ when comparing models fit to norovirus data in Berlin stratified by regions and age groups—models fit to the finest scales and subsequently upscaled had sharper predictions than models fit to the aggregated scales directly. This suggests that continued stratification of ILI, such as partitioning state-level ILI by age groups, by flu strain type, or county-level, may provide further sharpening of forecasts at aggregate scales.

Dante’s first place finish in the 2018/19 FluSight challenge may come as a surprise given that it is a purely statistical model and uses only ILINet data, while many of its competitors are based in full or in part on mechanistic disease transmission models and/or are augmented with alternative data sources (e.g., Google search data). Dante’s superior performance suggests that these mechanistic components or alternative data sources may be integrated into those models in a way that is improperly aligned with the truth. For example, DBM includes an SIR model component via a season-specific “I” term but a given season may have multiple circulating influenza strains responsible for the “true” flu component in the ILI data, thus rendering the use of a single “I” term inappropriate.

Revisions made to ILI data after its initial release are referred to as backfill and constitute a meaningful source of uncertainty when prospectively forecasting ILI. In this work, backfill was ignored for the forecasting of both Dante and DBM. As a result, the forecasting results for Dante and DBM are directly comparable to each other in this paper but are not directly comparable to previous FluSight challenge results. The reason backfill was ignored in this paper is because Dante uses state-level ILI data directly, and state-level backfill data is only available starting for the 2017/18 season. Though backfill was not addressed in this paper, Dante’s winning 2018/19 FluSight challenge entry did include a backfill model to account for the revision process of real-time ILI data.

Linking understandable processes to observed patterns in the data via models while maintaining high performance is the next frontier in ILI modeling. To do so will require a fuller consideration of the “ILI data generating process.” This process non-exhaustively includes a disease transmission process(es) (e.g., things often modeled with a compartmental model(s)), a healthcare visitation process (i.e., a set of processes related to who interacts with the healthcare system and when), an ILINet participation process (i.e., certain providers participate in ILINet while others do not, and the composition of provider networks varies temporally and spatially), and a reporting process (e.g., backfill).

Further stratification is a promising direction for incorporating known facets of the ILI data generating process into Dante in a flexible way. For example, if provider-level ILINet data were available state-level models could be decomposed into models for emergency department (ED) ILI and non-ED ILI. We hypothesize that a systematic difference exists between patients visiting ED and non-ED providers, specifically that the proportion of ED patients with ILI is higher than that of non-ED patients. If so, then provider composition could help explain some state-level variation in ILI magnitude (we expect states with more ED providers have higher reported ILI). It could also explain part of the holiday-specific spikes in observed ILI (we expect that spikes in ILI activity on holidays are partially due to the provider composition in ILINet changes for that week—more clinics are closed and thus ED providers have a relatively higher contribution).

Modeling across geographic scales in a single, unified model ensures forecasts are simultaneously coherent, a feature that is not present in many FluSight submissions. Ongoing work by our team will provide a model-agnostic tool by which users can modify outputs from a non-unified model so as to attain coherency^30^. Our team is also working to incorporate internet data sources (i.e., nowcasting) into future iterations of Dante. When internet data sources were incorporated into DBM the performance increased, which leads us to be hopeful that Dante will also be improved by the thoughtful incorporation of internet data.

While Dante has utility forecasting seasonal influenza, it would not be particularly useful for forecasting in an emerging outbreak setting. At its core, Dante is learning exploitable structure from data of historical flu seasons. An emerging outbreak, on the other hand, would by its very nature not have sufficient training data from which to learn about expected baseline trajectories. Swapping out the common process model term (${\mu }_{t}^{\,\text{all}\,}$– see the Methods section) for something resembling the infectious compartment of an SIR model might be one way to steer Dante towards emerging outbreak settings, but Dante’s core strength is its ability to exploit historical data.

## Methods

Dante is a probabilistic, Bayesian flu forecasting model that is decomposed into two submodels: the fine-scale model (i.e., the state model) and the aggregation model (i.e., the regional and national model).

### Dante’s fine-scale model

Dante’s fine-scale model is itself described in two parts: the data model and the process model.

#### Dante’s data model

Let *y*_rst_ ∈ (0, 1) be ILI/100 for state *r* = 1, 2, …, *R* in flu season *s* = 1, 2, …, *S*, for epidemic week *t* = 1, 2, …, *T* = 35, where *t* = 1 corresponds to epidemic week 40, roughly the first week of October and *T* = 35 most often corresponds to late May. Dante models the observed proportion *y*_rst_ with a Beta distribution as follows:

1 $${y}_{{\mathrm{rst}}}| {\theta }_{{\mathrm{rst}}},{\lambda }_{{\mathrm{r}}} \sim \,\text{Beta}\,({\lambda }_{{\mathrm{r}}}{\theta }_{{\mathrm{rst}}},{\lambda }_{{\mathrm{r}}}(1-{\theta }_{{\mathrm{rst}}})),$$

where

2 $$\,\text{E}\,({y}_{{\mathrm{rst}}}| {\theta }_{{\mathrm{rst}}},{\lambda }_{{\mathrm{r}}})={\theta }_{{\mathrm{rst}}},$$

3 $$\,\text{Var}\,({y}_{{\mathrm{rst}}}| {\theta }_{{\mathrm{rst}}},{\lambda }_{{\mathrm{r}}})=\frac{{\theta }_{{\mathrm{rst}}}(1-{\theta }_{{\mathrm{rst}}})}{1+{\lambda }_{{\mathrm{r}}}}.$$

In Dante, *θ*_rst_ is the unobserved true proportion of visits for ILI in state r for season *s* during week t and *λ*_r_ > 0 is a state-specific parameter that captures the level of noise in the ILINet surveillance system and thus the level of volatility in the ILI time series. In Dante, *y*_rst_ is modeled as unbiased for the latent state *θ*_rst_. The observation *y*_rst_, however, is not equal to *θ*_rst_ due to variability in the measurement surveillance process (i.e., the true proportion of ILI in state r for season s during week t is not going to be perfectly captured by ILINet surveillance). Motivated by Fig. 3, *λ*_r_ is likely to be related to ILINet participation as measured by the total number of patients seen weekly in state r. As *λ*_r_ increases, the variance of *y*_rst_ decreases and observations will tend to be closer to *θ*_rst_. Because we do not know the relationship between patient count and *λ*_r_ a priori, we model *λ*_r_ hierarchically, allowing them to be learned from data (details in Supplementary Note 1.1). Specifically, each *λ*_r_ is given a central, non-standardized t-distribution prior with support in the interval [0, *∞*) and a shared precision parameter that itself has a weakly informative Gamma prior.

**Fig. 8:**
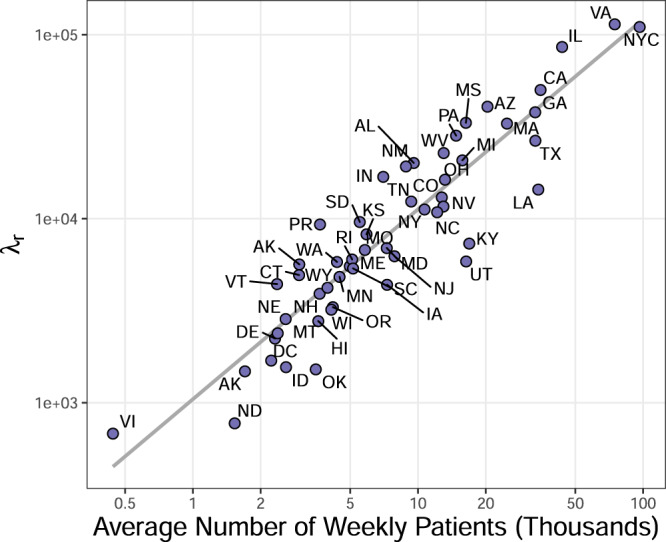
Dante’s posterior means for *λ*_r_. The posterior mean for *λ*_r_ versus the average number of patients seen weekly by each state. Both axes are on a log scale. A clear linear relationship is observed. Dante learns this relationship, as it has no explicit knowledge of the average number of patients.

Figure 8 shows the posterior mean of *λ*_r_ versus the average number of patients seen weekly by each state. A clear linear relationship is observed on a log-log scale, where the variance of *y*_rst_ goes down (i.e., *λ*_r_ increases) as the total weekly seen patients increases. What is particularly striking about Fig. 8 is that Dante has no knowledge of the number of patients seen each week as it is not an input to Dante, illustrating how structure can be learned rather than prescribed with a flexible, hierarchical model, provided sufficient training data.

#### Dante’s Process Model

Dante’s process model models the unobserved true proportion of ILI, *θ*_rst_ ∈ (0, 1), as a function of four components:

4 $${\theta }_{{\mathrm{rst}}}={\text{logit}}^{-1}({\pi }_{{\mathrm{rst}}}),$$

5 $${\pi }_{{\mathrm{rst}}}={\mu }_{{\mathrm{t}}}^{\,\text{all}}+{\mu }_{{\mathrm{rt}}}^{\text{state}}+{\mu }_{{\mathrm{st}}}^{\text{season}}+{\mu }_{{\mathrm{rst}}}^{\text{interaction}\,}.$$

The two season independent terms in Eq. (5), ${\mu }_{{\mathrm{t}}}^{\,\text{all}\,}$ and ${\mu }_{{\mathrm{rt}}}^{\,\text{state}\,}$, are modeled as random walks, and the two season dependent terms, ${\mu }_{{\mathrm{st}}}^{\,\text{season}\,}$ and ${\mu }_{{\mathrm{rst}}}^{\,\text{interaction}\,}$, are modeled as reverse-random walks. Random and reverse-random walks allow patterns in the process model to be flexibly learned while capturing week-to-week correlation. Standard random walk priors are used for ${\mu }_{{\mathrm{t}}}^{\,\text{all}\,}$ and ${\mu }_{{\mathrm{rt}}}^{\,\text{state}\,}$, with the latter being specified hierarchically. This specification involves placing a mean-0 normal prior on the first week of the flu season, and assuming a priori that subsequent weeks are normally distributed and centered at the previous week’s value. The reverse random walk term ${\mu }_{{\mathrm{st}}}^{\,\text{season}\,}$ requires a prior specification for the value in the final week of the flu season rather than the first week of the season, which allows the forecasting extrapolation problem to become an interpolation problem. The state-season specific term ${\mu }_{{\mathrm{rst}}}^{\,\text{interaction}\,}$ is the most challenging for Dante to learn because it is only directly informed by state *r* and season *s*, and for forecasting purposes there is either no or partial information available from season *s*. For that reason, an autoregressive term is included in the hierarchical reverse random walk prior specification to help regularize ${\mu }_{{\mathrm{rst}}}^{\,\text{interaction}\,}$ towards 0. Prior choice is an aspect of the model which could still be improved, and it is possible that alternative prior specifications may yield improved performance. More details are available in Supplementary Note 1.2.

**Fig. 9:**
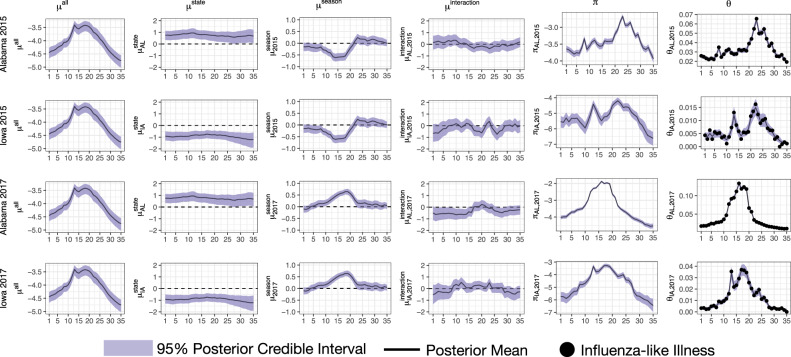
Posterior summaries of Dante’s process model. Posterior summaries for select components, seasons, and states of Dante, fit to seasons 2010 through 2017. Rows, from top to bottom, correspond to Alabama in 2015, Iowa in 2015, Alabama in 2017, and Iowa in 2017. Columns, from left to right, correspond to *μ*^all^, *μ*^state^, *μ*^season^, *μ*^interaction^, *π* (all from Equation (5)), and *θ* (Equation (4)). The *π* column is the sum of the *μ*^all^, *μ*^state^, *μ*^season^, and *μ*^interaction^ columns, accounting for posterior covariances. The *θ* column is the inverse logit of the *π* column and is back on the scale of the data. The *μ*^all^ component is the most structured component, as it is common for all states and seasons (i.e., the same for all rows). The components *μ*^state^ and *μ*^season^ are the next most structured components. They describe the state-specific and season-specific deviations from *μ*^all^, respectively, and are common for all seasons within a state (*μ*^state^) and all states within a season (*μ*^season^). The component *μ*^interaction^ is the least structured component of Dante, as it is specific to each season/state (i.e., it is different for each row). Solid lines are posterior means. Ribbons are 95% posterior intervals. In the *θ* column, points are data, *y*.

Figure 9 illustrates the fits for all model components for Alabama and Iowa for the 2015 and 2017 flu seasons. The component ${\mu }_{{\mathrm{t}}}^{\,\text{all}\,}$ is common to every state and season and acts as the anchor for the process model. The shape of ${\mu }_{{\mathrm{t}}}^{\,\text{all}\,}$ is similar to the national average ILI trajectory of Fig. 1, capturing the profile for a typical state and season. The component ${\mu }_{{\mathrm{rt}}}^{\,\text{state}\,}$ captures the state-specific deviation from ${\mu }_{{\mathrm{t}}}^{\,\text{all}\,}$ and is common to every season for a given state, but is distinct for each state. As can be see in Fig. 1, Alabama typically sees ILI above the national average, hence why ${\mu }_{{\mathrm{rt}}}^{\,\text{state}\,}$ for Alabama is learned to be greater than zero. Iowa, however, typically sees ILI below the national average, explaining why ${\mu }_{{\mathrm{rt}}}^{\,\text{state}\,}$ is learned to be less than zero for Iowa. The component ${\mu }_{{\mathrm{st}}}^{\,\text{season}\,}$ captures the season-specific deviation from ${\mu }_{{\mathrm{t}}}^{\,\text{all}\,}$ and is common to every state for a given season, but is distinct for each season. This component captures the fact that seasons can have effects that are shared by nearly all states, as illustrated in Fig. 2. The shape of ${\mu }_{{\mathrm{st}}}^{\,\text{season}\,}$ for 2015 and 2017 has a similar shape to the average residuals for 2015 and 2017, respectively, in Fig. 2. Finally, ${\mu }_{{\mathrm{rst}}}^{\,\text{interaction}\,}$ captures the remaining signal in *π*_rst_ that cannot be accounted for by ${\mu }_{{\mathrm{t}}}^{\,\text{all}\,}$, ${\mu }_{{\mathrm{rt}}}^{\,\text{state}\,}$, and ${\mu }_{{\mathrm{st}}}^{\,\text{season}\,}$. The term ${\mu }_{{\mathrm{rst}}}^{\,\text{interaction}\,}$ is distinct for each state and season.

Dante’s process model is purposely over-specified. If our interest were purely to fit ILI data, the term ${\mu }_{{\mathrm{rst}}}^{\,\text{interaction}\,}$ alone would suffice. However, there is not enough structure to forecast effectively with only ${\mu }_{{\mathrm{rst}}}^{\,\text{interaction}\,}$. On the other hand, the non-interaction terms in the decomposition of *π*_rst_ (${\mu }_{{\mathrm{t}}}^{\,\text{all}\,}$, ${\mu }_{{\mathrm{rt}}}^{\,\text{state}\,}$, and ${\mu }_{{\mathrm{st}}}^{\,\text{season}\,}$) provide structure for forecasting but not enough flexibility to capture all the signal in the ILI data. Thus, the ${\mu }_{{\mathrm{rst}}}^{\,\text{interaction}\,}$ term provides the flexibility needed to fit the data, but is specified so that it plays as minimal a role as possible so that signal is captured in the non-interaction terms and can drive the shape of forecasts.

Inference for unobserved components of Dante, as well as state-level forecasts of yet-to-be-observed *y*_rst_ are generated by sampling from the posterior distribution with Markov chain Monte Carlo (MCMC), resulting in a sample of *M* draws that summarize the posterior distribution (details in Supplementary Note 2). We use the software JAGS (Just Another Gibbs Sampler)^31^, as called by the R package rjags^32^ within the programming language R^33^ to perform the MCMC sampling. We denote each MCMC draw by the index *m*. Notationally, we denote the *m*th sample for a yet-to-be-observed *y*_rst_ as *y*_rstm_.

### Dante’s aggregation model

Dante’s regional and national forecasts are computed as linear combinations of state forecasts, where weights are proportional to 2010 US Census population estimates (Supplementary Fig. 6). Let *w*_r_ ∈ [0, 1] be the population of state r divided by the US population such that $\mathop{\sum }\nolimits_{r = 1}^{R}{w}_{r}=1$. For each MCMC draw *m*, we compute the ILI forecast for aggregated region *ρ* (indexing all ten HHS regions and nationally) as:

6 $${y}_{\rho {\mathrm{stm}}}=\mathop{\sum }\limits_{r=1}^{R}{w}_{{\mathrm{r}}}^{(\rho)}{y}_{{\mathrm{rstm}}}.$$

For *ρ* = region X (e.g., *ρ* = HHS Region 1), ${w}_{r}^{(\rho)}=0$ if state *r* is not a member of region X and

7 $${w}_{r}^{(\rho)}=\frac{{w}_{r}\,\text{I}\,(r\in \,\text{region X}\,)}{\mathop{\sum }\nolimits_{r = 1}^{R}{w}_{r}\,\text{I}\,(r\in \,\text{region X}\,)}$$

and I(*r* ∈ region X) is an indicator function equal to 1 if state *r* is in region X and 0 otherwise. By construction, $\mathop{\sum }\nolimits_{r = 1}^{R}{w}_{r}^{(\rho)}=1$ for any *ρ*. The aggregation model constitutes a bottom-up forecasting procedure and ensures forecasts are coherent across scales. An example of resulting aggregated forecasts can be found in Supplementary Note 7.

### Reporting Summary

Further information on research design is available in the Nature Research Reporting Summary linked to this article.

## Supplementary information

Supplementary Information

Reporting Summary

## Data Availability

Up-to-date national, regional, and state ILI data are available from the CDC’s FluView Interactive application: https://gis.cdc.gov/grasp/fluview/fluportaldashboard.html. Select “ILINet” as the data source, “National,” “HHS Regions,” and “State” as the regions, and “Select All” for the seasons.

## Code availability

The code to run Dante has been provided in the Supplementary Information document. A minimum working example of the code can be found here: https://github.com/lanl/Dante.
