# Supplementary material for: Multiscale influenza forecasting
Source: Nat Commun. 2021 May 20;12:2991. doi: 10.1038/s41467-021-23234-5 (PMC8137955; doi:10.1038/s41467-021-23234-5)
Supplement: Supplementary file 1 — Supplementary Information [file 41467_2021_23234_MOESM1_ESM.pdf]

Supplemental Information:
Multiscale Influenza Forecasting

Dave Osthus*[a] and Kelly R. Moran[a,b]

[a]Los Alamos National Laboratory, Statistical Sciences Group

[b]Duke University, Department of Statistical Science

*Email correspondence can be directed to dosthus@lanl.gov

Supplementary Note 1. Dante's state-level model

Dante's state-level model is a Bayesian, hierarchical model composed of hierarchically specified random and reverse random walks. Dante's state-level model is decomposed into a process model and a data model specified conditionally on the process model. In what follows, we describe all the modeling details of Dante's state-level model.

Throughout Dante's specification, pragmatic prior distributional and hyperparameter choices were made that we believe are, on balance, reasonable. We feel justified in this position, given Dante's winning performance in the CDC's prospective 2018/19 FluSight challenge. That said, it is possible further improvements to Dante's forecasting performance could be achieved through a more rigorous investigation into choices of priors and hyperparameters. A rigorous investigation could include performing formal cross-validation over combinations of prior distributions and hyperparameters.

Supplementary Note 1.1. Data model

Let $y_{rst} \in (0,1)$ represent observed influenza-like illness (ILI) as a proportion for state $r = 1, 2, \ldots, R$, for flu season $s = 1, 2, \ldots, S$, and week of season $t = 1, 2, \ldots, T$. Dante models ILI as

*Email correspondence can be directed to dosthus@lanl.gov

$$y_{rst}|\theta_{rst}, \lambda_r \sim \text{Beta}(\lambda_r \theta_{rst}, \lambda_r(1 - \theta_{rst})), \tag{1}$$

where $\theta_{rst} \in (0, 1)$. That is, observed ILI/100 is modeled with a Beta distribution where

$$\text{E}(y_{rst}|\theta_{rst}, \lambda_r) = \theta_{rst}, \tag{2}$$

$$\text{Var}(y_{rst}|\theta_{rst}, \lambda_r) = \frac{\theta_{rst}(1 - \theta_{rst})}{1 + \lambda_r}. \tag{3}$$

The conditional variance of y_{rst} is modeled with a state-specific parameter $\lambda_r > 0$, allowing different states to have different variances. The data y_{rst} is a proportion, where the denominator of the proportion is the number of patient visits reported by ILINet providers for any reason. Different states have widely different volumes of patient visits reported by ILINet, ranging from less than 1000 a week by the US Virgin Islands to just under 100,000 a week in Virginia and New York City. The noise in those estimates is related to those patient visits, with noisier estimates associated with smaller patient visit volumes. The noise in the estimated proportions are captured by the parameter λ_r, as illustrated in Supplementary Figure 1. As λ_r decreases, the variance of the Beta distribution increases, resulting in noisier realizations from the data model.

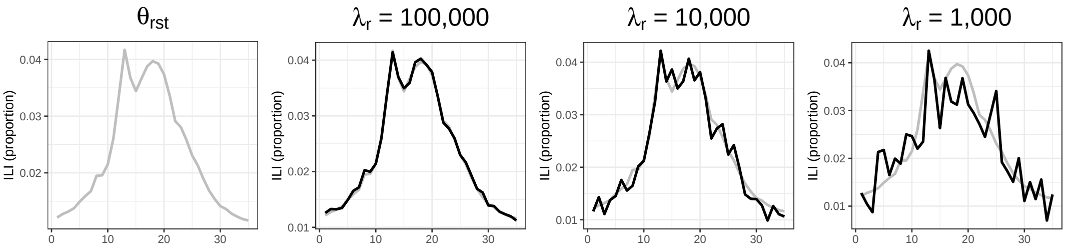

Supplementary Figure 1: The underlying mean of Dante's data model (left) and three realizations from Dante's data model under three different values of λ_r. When λ_r is large, realizations (black line) closely match the underlying mean process (grey line). As λ_r gets smaller, realizations from the data model deviate more significantly from the underlying mean process.

The parameter λ_r, i.e. the state-specific variance parameter, is modeled hierarchically

$$\lambda_r|\lambda_{\text{prec}} \sim \text{t}_{[0,\infty)}(0, \lambda_{\text{prec}}, 3), \tag{4}$$

$$\lambda_{\text{prec}} \sim \text{Gamma}(5, 5), \tag{5}$$

where $\text{t}_{[0,\infty)}$ is a half, non-standardized t-distribution with support in the interval $[0, \infty)$. The degrees of freedom parameter is set to be 3 to ensure that the mean and variance for the non-standardized t-distribution are defined while still maintaining a heavy-tailed distribution. The

non-centrality parameter is set to 0, corresponding to a central, non-standardized t-distribution. The Gamma(5,5) prior for λ_{prec} was selected as a weakly informative prior with prior expectation for λ_{prec} equal to 1. The goal of this prior is to provide mild regularization, giving unlikely parameter values low prior weight while not *a priori* ruling out any positive values for λ_{prec}. A non-standardized t-distribution with non-centrality parameter equal to 0 and $\lambda = 1$ corresponds to the Student's t-distribution with only a degrees of freedom parameter. The parameter λ_r was not modeled explicitly as a function of number of patient visits as 1) the form of the relationship between λ_r and patient visit volume was not known *a priori* and 2) the number of future patient visits are not available for forecasting purposes.

Supplementary Note 1.2. Process model

The process model is a model for the quantity θ_{rst}, the latent expected ILI proportion for state r in season s for week of season t. Dante model's θ_{rst} as

$$\theta_{rst} = \text{logit}^{-1}(\pi_{rst}), \tag{6}$$

$$\pi_{rst} = \mu_t^{\text{all}} + \mu_{rt}^{\text{state}} + \mu_{st}^{\text{season}} + \mu_{rst}^{\text{interaction}}. \tag{7}$$

That is, Dante models θ_{rst} as a function of the sum of four components: μ_t^{all}, μ_{rt}^{state}, μ_{st}^{season}, and $\mu_{rst}^{\text{interaction}}$. The models for those components are subsequently described.

Supplementary Note 1.2.1. Process model: μ_t^{all}

The term μ_t^{all} is the anchor of the process model. It captures structure across the flu season common to all states and seasons. Specifically, μ_t^{all} is modeled as a random walk. For $t = 1$,

$$\mu_1^{\text{all}} | \sigma_1^{2,\text{all}} \sim \text{N}(0, \sigma_1^{2,\text{all}}), \tag{8}$$

$$\sigma_1^{2,\text{all}} | \tau_1^{\text{all}} \sim \text{N}_{[0,\infty)}(0, 1/\tau_1^{\text{all}}), \tag{9}$$

$$\tau_1^{\text{all}} \sim \text{Gamma}(5, 5), \tag{10}$$

where $\text{N}_{[0,\infty)}$ is a half Normal distribution. For $t = 2, 3, \ldots, T$,

$$\mu_t^{\text{all}} | \mu_{t-1}^{\text{all}}, \sigma^{2,\text{all}} \sim \text{N}(\mu_{t-1}^{\text{all}}, \sigma^{2,\text{all}}), \tag{11}$$

$$\sigma^{2,\text{all}} | \tau^{\text{all}} \sim \text{N}_{[0,\infty)}(0, 1/\tau^{\text{all}}), \tag{12}$$

$$\tau^{\text{all}} \sim \text{Gamma}(5, 5). \tag{13}$$

Supplementary Note 1.2.2. Process model: μ_{rt}^{state}

The term μ_{rt}^{state} captures the state-specific deviation from μ_t^{all}, capturing effects common to all seasons within a state but distinct across states. The term μ_{rt}^{state} is modeled as a hierarchical random walk.

For $r = 1, 2, \ldots, R$, $\mu_{r,1}^{\text{state}}$ is modeled as

$$\mu_{r,1}^{\text{state}} | \sigma_0^{2,\text{state}} \sim \mathrm{N}(0, \sigma_0^{2,\text{state}}), \tag{14}$$

$$\sigma_0^{2,\text{state}} | \tau_0^{\text{state}} \sim \mathrm{N}_{[0,\infty)}(0, 1/\tau_0^{\text{state}}), \tag{15}$$

$$\tau_0^{\text{state}} \sim \mathrm{Gamma}(5, 5). \tag{16}$$

For $r = 1, 2, \ldots, R$ and $t = 2, 3, \ldots, T$,

$$\mu_{rt}^{\text{state}} | \mu_{r,t-1}^{\text{state}}, \sigma_r^{2,\text{state}} \sim \mathrm{N}(\mu_{r,t-1}^{\text{state}}, \sigma_r^{2,\text{state}}). \tag{17}$$

For $r = 1, 2, \ldots, R$,

$$\sigma_r^{2,\text{state}} | \lambda^{\text{state}} \sim \mathrm{t}_{[0,\infty)}(0, \lambda^{\text{state}}, 3), \tag{18}$$

with

$$\lambda^{\text{state}} \sim \mathrm{Gamma}(5, 5). \tag{19}$$

Supplementary Note 1.2.3. Process model: μ_{st}^{season}

The term μ_{st}^{season} captures the season-specific deviation from μ_t^{all}, capturing effects common to all states within a season but distinct across seasons. The term μ_{st}^{season} is modeled as a reverse random walk. The reverse random walk requires a prior specification for the final week of the season, T, rather than the first week of the season as is required with a random walk. Terms indexed by season s are prone to large uncertainty intervals when used for forecasting, as no data are available to constrain their trajectories. By modeling μ_{st}^{season} as a reverse random walk and placing a prior on time T, we effectively turn our forecasting extrapolation problem into an interpolation problem. Though the flu season is highly variable in the middle of the season, it is quite well-constrained

at the end of the season (e.g., the end of May). This makes specifying a prior at the end of the flu season a safer proposition than it may at first appear.

For $s = 1, 2, \ldots, S$, the model for μ_{sT}^{season} is

$$\begin{aligned}
\mu_{sT}^{\text{season}} | \sigma_T^{2,\text{season}} &\sim \text{N}(0, \sigma_T^{2,\text{season}}), && (20)\\
\sigma_T^{2,\text{season}} | \lambda^{\text{season}} &\sim \text{t}_{[0,\infty)}(0, \lambda^{\text{season}}, 3), && (21)\\
\lambda^{\text{season}} &\sim \text{Gamma}(5, 5). && (22)
\end{aligned}$$

For $s = 1, 2, \ldots, S$ and for $t = 1, 2, \ldots, T-1$,

$$\begin{aligned}
\mu_{st}^{\text{season}} | \mu_{s,t+1}^{\text{season}}, \sigma^{2,\text{season}} &\sim \text{N}(\mu_{s,t+1}^{\text{season}}, \sigma^{2,\text{season}}), && (23)\\
\sigma^{2,\text{season}} | \sigma_T^{2,\text{season}}, \lambda^{\text{season}} &\sim \text{t}_{[0,\sigma_T^{2,\text{season}}]}(0, \lambda^{\text{season}}, 3). && (24)
\end{aligned}$$

The model for $\sigma^{2,\text{season}}$ is constrained to be less than $\sigma_T^{2,\text{season}}$ helping constrain the trajectories of μ_{st}^{season} from wandering wildly.

Supplementary Note 1.2.4. Process model: $\mu_{rst}^{\textbf{interaction}}$

The term $\mu_{rst}^{\text{interaction}}$ captures the season-specific deviation from $\mu_t^{\text{all}} + \mu_{rt}^{\text{state}} + \mu_{st}^{\text{season}}$, capturing structure that is specific to season s and state r. Dante models $\mu_{rst}^{\text{interaction}}$ as a hierarchical reverse random walk.

For $r = 1, 2, \ldots, R$ and $s = 1, 2, \ldots, S$, $\mu_{rsT}^{\text{interaction}}$ is modeled as

$$\begin{aligned}
\mu_{rsT}^{\text{interaction}} | \eta_r^{\text{interaction}}, \sigma_{rT}^{2,\text{interaction}} &\sim \text{N}(\eta_r^{\text{interaction}}, \sigma_{rT}^{2,\text{interaction}}), && (25)\\
\eta_r^{\text{interaction}} | \sigma_0^{2,\text{interaction}} &\sim \text{N}(0, \sigma_0^{2,\text{interaction}}), && (26)\\
\sigma_0^{2,\text{interaction}} &\sim \text{N}_{[0,\infty)}(0, 20), && (27)
\end{aligned}$$

where the variance of 20 was selected as a wide, uninformative prior for $\sigma_0^{2,\text{interaction}}$. For $r = 1, 2, \ldots, R$, $s = 1, 2, \ldots, S$ and $t = 1, 2, \ldots, T-1$,

$$\mu_{rst}^{\text{interaction}} | \mu_{rs,t+1}^{\text{interaction}}, \alpha_r^{\text{interaction}}, \sigma_{rt}^{2,\text{interaction}} \sim \text{N}(\alpha_r^{\text{interaction}} \mu_{rs,t+1}^{\text{interaction}}, \sigma_{rt}^{2,\text{interaction}}). \quad (28)$$

The autoregressive term $\alpha_r^{\text{interaction}} \in (0,1)$ helps regularize $\mu_{rst}^{\text{interaction}}$ towards 0. Because $\mu_{rst}^{\text{interaction}}$ is modeled as a reverse-random walk, as we step backward in time from T to $T-1$ all the way to current time t (i.e., T minus some $k \geq 0$), the shrinking effect on

$$\text{E}(\mu_{rst}^{\text{interaction}} | \mu_{rs,t+1}^{\text{interaction}}, \alpha_r^{\text{interaction}}, \sigma_{rt}^{2,\text{interaction}})$$

due to $\alpha_r^{\text{interaction}} \in (0,1)$ increases with distance from T (i.e., with increasing k). Specifically, by the law of iterated expectations and noting all expectations are implicitly conditioned on $\eta_r^{\text{interaction}}$, $\alpha_r^{\text{interaction}}$, and $\sigma_{rt}^{2,\text{interaction}}$ for $t = 1, 2, \ldots, T$, we have the following:

$$\begin{aligned}
\text{E}(\mu_{rsT}^{\text{interaction}}) &= \eta_r^{\text{interaction}}, && \text{(Equation (25))} \\
\text{E}(\mu_{rs,T-1}^{\text{interaction}}) &= \text{E}(\text{E}(\mu_{rs,T-1}^{\text{interaction}} | \mu_{rsT}^{\text{interaction}})) \\
&= \text{E}(\alpha_r^{\text{interaction}} \mu_{rsT}^{\text{interaction}}) && \text{(Equation (28))} \\
&= \alpha_r^{\text{interaction}} \text{E}(\mu_{rsT}^{\text{interaction}}) \\
&= \alpha_r^{\text{interaction}} \eta_r^{\text{interaction}}, \\
&\vdots \\
\text{E}(\mu_{rs,T-k}^{\text{interaction}}) &= \text{E}(\text{E}(\mu_{rs,T-k}^{\text{interaction}} | \mu_{rs,T-(k-1)}^{\text{interaction}})) \\
&= \text{E}(\alpha_r^{\text{interaction}} \mu_{rs,T-(k-1)}^{\text{interaction}}) \\
&= \alpha_r^{\text{interaction}} \text{E}(\mu_{rs,T-(k-1)}^{\text{interaction}}) \\
&\vdots \\
&= (\alpha_r^{\text{interaction}})^k \text{E}(\mu_{rsT}^{\text{interaction}}) \\
&= (\alpha_r^{\text{interaction}})^k \eta_r^{\text{interaction}}.
\end{aligned}$$

We found this regularization helps the forecast intervals from getting too large, as $\mu_{rst}^{\text{interaction}}$ is the most challenging term in Dante to learn as only data from the state r and season s directly constrain it. For forecasting purposes, we have no or only partial data available for season s. In general, more care needs to be taken to model and constrain components indexed by s, as there will be little data to constrain those terms when Dante is used for forecasting.

For $r = 1, 2, \ldots, R$, the model for the autoregressive term $\alpha_r^{\text{interaction}}$ is,

$$\alpha_r^{\text{interaction}} | \nu_{\text{a}}^{\text{interaction}}, \nu_{\text{b}}^{\text{interaction}} \sim \text{Beta}(\nu_{\text{a}}^{\text{interaction}}, \nu_{\text{b}}^{\text{interaction}}), \tag{29}$$

$$\nu_{\text{a}}^{\text{interaction}} \sim \text{Gamma}(5,5), \tag{30}$$

$$\nu_{\text{b}}^{\text{interaction}} \sim \text{Gamma}(5,5). \tag{31}$$

The prior means for $\nu_{\text{a}}^{\text{interaction}}$ and $\nu_{\text{a}}^{\text{interaction}}$ are both 1. A Beta(1,1) is a Uniform(0,1) distribution. Thus, the prior choices for $\alpha_r^{\text{interaction}}$ reflect a lack of prior information for the autoregressive term $\alpha_r^{\text{interaction}}$.

Finally, for $r = 1, 2, \ldots, R$ and $t = 1, 2, \ldots, T$,

$$\sigma_{rt}^{2,\text{interaction}} | \lambda^{\text{interaction}} \sim \text{t}_{[0,\infty)}(0, \lambda^{\text{interaction}}, 3), \tag{32}$$

$$\lambda^{\text{interaction}} \sim \text{Gamma}(5,5). \tag{33}$$

Supplementary Note 2. MCMC and code

Samples from the posterior distributions and posterior predictive distributions of Dante are drawn via Markov chain Monte Carlo (MCMC). We use the software JAGS (Just Another Gibbs Sampler) as called from the `rjags` package in `R`. JAGS requires the model be specified as a directed acyclic graph, hence why the model in Supplementary Note 1 is conditionally specified. We run three MCMC chains. Each chain is run for 30,000 iterations, keeping every 10th iteration, resulting in 3,000 draws per chain after thinning. We discard the first 1,500 thinned draws as burnin, resulting in 1,500 samples per chain, or a total of M = 4,500 samples per JAGS run.

Supplementary Note 2.1. JAGS code

The following quantities are passed into JAGS:

- `NT` is a scalar indicating how many weeks are to be modeled in the flu season. It is set equal to 35.
- `NR_state` is a scalar for the number of states to be modeled. It is set equal to 54, including Florida.
- `NR_region` is a scalar for the number of HHS regions to be modeled. It is set equal to 10.
- `NS` is a scalar for the number of flu seasons to be modeled. It is set equal to 8, corresponding to the 8 seasons inclusively between the 2010/11 and 2017/18 seasons.

- `nobs` is a scalar denoting how many weeks of the flu season have been observed at the time of forecasting. It is set to an integer between 5 and 29, inclusively.
- `fcstseason` is a scalar denoting the index for the flu season being forecasted. It is set to an integer between 3 and 8, inclusively. Due to missing data, we do not forecast seasons 2010/11 and 2011/12. However, the available data for those two seasons are used for fitting.
- `yobs_nat` is a vector of length `nobs` with the national wILI/100 estimates corresponding to the first `nobs` weeks of the `fcstseason` flu season.
- `yobs_region` is an `nobs` × `NR_region` matrix where each row corresponds to a week of the flu season and each column corresponds to an HHS Region. Entry `yobs_region[t,r]` is the wILI/100 estimate corresponding to week `t` of flu season `fcstseason` for HHS Region `r`.
- `yobs_state` is an `NR_state` × `NS` × `nobs` dimensional array where the first dimension indexes states, the second dimension indexes flu season, and the third dimension indexes week of flu season. The `yobs_state[r,fcstseason,t]` entry is the ILI/100 estimate for state `r` of flu season `fcstseason` of week of flu season `t`.
- `y_state` is an `NR_state` × `NS` × `NT` dimensional array where the first dimension indexes states, the second dimension indexes flu season, and the third dimension indexes week of flu season. The `y_state[r,s,t]` entry is the ILI/100 estimate for state `r` of flu season `s` of week of flu season `t`. If the ILI/100 estimate is missing for index `[r,s,t]`, the missing value indicator `NA` is used. When ILI/100 is very close to zero, JAGS struggles with numerical stability issues. Thus, for all non-missing ILI/100 values, we set all ILI/100 less than 0.0005 to 0.0005.
- `census_weights` is an `NR_state` × `NR_region+1` matrix where each column sums to 1 and each entry is greater than or equal to 0. The first `NR_region` columns correspond to the `NR_region` HHS Regions. The last column corresponds to the nation. Each row corresponds to a state. The `census_weights[r,i]` entry is the relative weight state `r` has in geographic region `i`, as determined by 2010 US Census population estimates. If the entry `census_weights[r,i]` equals 0, that means state `r` is not in geographic region `i`.

The JAGS code for Dante, state-level model and aggregation model, is below.

```
model{

  ##############################################
  ## AGGRETATION MODEL

  ##############################################
  ## national forecast model
```

```
for(t in (nobs+1):NT){
  futurey_nat[t] <- t(census_weights[,NR_region+1]) %*% futurey_state[,t]
}
for(t in 1:nobs){
  futurey_nat[t] <- yobs_nat[t]
}

#############################################
## region forecast model
for(r in 1:NR_region){
  for(t in (nobs+1):NT){
    futurey_hhs[r,t] <- t(census_weights[,r]) %*% futurey_state[,t]
  }
}
for(r in 1:NR_region){
  for(t in 1:nobs){
    futurey_hhs[r,t] <- yobs_region[t,r]
  }
}

#############################################
## draw from state-level posterior predictive distribution
for(r in 1:NR_state){
  for(t in (nobs+1):NT){
    futurey_state[r,t] ~ dbeta(lambda[r] * pi_all[r,fcstseason,t],
                               lambda[r] * (1 - pi_all[r,fcstseason,t]))
  }
}
for(r in 1:NR_state){
  for(t in 1:nobs){
    futurey_state[r,t] <- yobs_state[r,fcstseason,t]
  }
}


##############################################
## DANTE'S DATA MODEL

##############################################
## state data model
for(r in 1:NR_state){
  for(s in 1:NS){
    for(t in 1:NT){

      ## data model
```

```
      yobs_state[r,s,t] ~ dbeta(lambda[r] * pi_all[r,s,t],
                               lambda[r] * (1 - pi_all[r,s,t]))

      ## data mean
      pi_all[r,s,t] <- ilogit(mu_all[t] + mu_season[s,NT+1-t] +
                                mu_state[r,t] + mu_interaction[r,s,NT+1-t])

    }
  }
}

for(r in 1:NR_state){
  lambda[r] ~ dt(0, lambda_prec, 3) T(0,)
}
lambda_prec ~ dgamma(5, 5)


###########################################
## DANTE'S PROCESS MODEL

###########################################
## mu_all time series
mu_all[1] ~ dnorm(0, pow(var_all_init, -1))

for(t in 2:NT){
  mu_all[t] ~ dnorm(mu_all[t-1], pow(var_all,-1))
}

var_all_init  ~ dnorm(0, prec_all_init) T(0,)
var_all       ~ dnorm(0, prec_all) T(0,)
prec_all_init ~ dgamma(5, 5)
prec_all      ~ dgamma(5, 5)


###########################################
## mu_season time series

for(s in 1:NS){
  mu_season[s,1] ~ dnorm(0, pow(var_season_init, -1))
}

for(s in 1:NS){
  for(t in 2:NT){
    mu_season[s,t] ~ dnorm(mu_season[s,t-1], pow(var_season, -1))
  }
```

```
}

var_season_init   ~ dt(0, prec_season, 3) T(0,)
var_season        ~ dt(0, prec_season, 3) T(0, var_season_init)
prec_season       ~ dgamma(5, 5)


###########################################
## mu_state time series

for(r in 1:NR_state){
  mu_state[r,1] ~ dnorm(0, pow(var_state_init, -1))
}

for(r in 1:NR_state){
  for(t in 2:NT){
    mu_state[r,t] ~ dnorm(mu_state[r,t-1], pow(var_state[r], -1))
  }
}
for(r in 1:NR_state){
  var_state[r] ~ dt(0, prec_state, 3) T(0,)
}
prec_state ~ dgamma(5, 5)

var_state_init  ~ dnorm(0, prec_state_init) T(0,)
prec_state_init ~ dgamma(5, 5)


###########################################
## mu_interaction time series

## mu_interaction initialization
for(r in 1:NR_state){
  for(s in 1:NS){
    mu_interaction[r,s,1] ~ dnorm(mu_interaction_mean[r], pow(var_interaction[r,1], -1))
  }
  mu_interaction_mean[r] ~ dnorm(0,pow(var_interaction_mean,-1))
}
var_interaction_mean ~ dnorm(0,pow(.05,-1)) T(0,)

for(r in 1:NR_state){
  for(s in 1:NS){
    for(t in 2:NT){
      mu_interaction[r,s,t]  ~  dnorm(alpha_interaction[r] * mu_interaction[r,s,t-1],
                                      pow(var_interaction[r,t], -1))
```

```
      }
    }
  }
  for(r in 1:NR_state){
    alpha_interaction[r] ~ dbeta(alpha_interaction_a, alpha_interaction_b)
  }
  alpha_interaction_a ~ dgamma(5, 5)
  alpha_interaction_b ~ dgamma(5, 5)


  ## prior over interaction variances
  for(r in 1:NR_state){
    for(t in 1:NT){
      var_interaction[r,t]  ~ dt(0, prec_interaction, 3) T(0,)
    }
  }
  prec_interaction ~ dgamma(5, 5)

}
```

Supplementary Note 2.2. System requirements and installation guide

As noted in the previous subsection, Dante uses the software JAGS (Just Another Gibbs Sampler) as called by the `R` package `rjags` within the programming language `R` to perform the MCMC sampling. The code is supported on all operating systems for which the requisite downloads (see below) are possible. The example code was tested on a 64-bit x86_64-redhat-linux-gnu platform running Fedora 29, using `R` version 3.6.1, `rjags` 4-10, and `coda` 0.19-3.

To download and install software and packages:

- `R` ($\geq$ 2.14.0) follow instructions at `https://www.r-project.org/`
- `rjags` ($\geq$ 4-10) run `install.packages('rjags')` within `R` session
- `coda` ($\geq$ 0.13) run `install.packages('coda')` within `R` session

Installation should take less than 15 minutes on a normal desktop computer.

Supplementary Note 2.3. Demonstration

A reduced data set is provided, named `dat.Rdata`. This example data includes ILI observations from epi seasons 2013, 2014, and 2015 for the states Alaska, Illinois, Indiana, Maryland, Ohio, Virginia, Washington, West Virginia, and Wisconsin. For 2013 and 2014, data from epi time 1

through 30 are included; for 2015, the ‘forecast season’, ILI is observed only from epi time 1 through 14. Note that ‘regional’ and ‘national’ data are also scaled down to this set of states.

The R code to collect samples from Dante using this data set is found below. Note that for the model to be constructed using the following snippet, users must first save the model code (found in Supplementary Note 2.1) as Dante.bug and then run the code in R. After model construction and sampling, the code creates a series of three plots: trace plots and density estimates for two of the state-specific precision parameters in the model, followed by the ‘national’ observed data with forecast mean and 95% credible interval. The code should take roughly 2-3 minutes to run.

```
# Load required library
library(’rjags’)

# Set path to Dante model code and data file
filepath = ’~/path/to/files/’

# Load data, which takes the form of a list.
load(paste0(filepath,’dat.Rdata’))

# Add small nonzero value to the ILI=0 entries
dat$yobs_state[dat$yobs_state==0] = 0.0005

# Compile the model.
mod <- jags.model(file = paste0(filepath,’Dante.bug’),
                  data = dat,
                  n.chains = 1,   # One chain used for illustrative purposes.
                  n.adapt = 1000) # Burn-in for 1000 iterations.

# Generate posterior samples from the model in mcmc.list format,
# specifying that values futurey_nat and lambda be saved.
res <- coda.samples(model = mod,
                    variable.names = c(’futurey_nat’,’lambda’),
                    n.iter = 2000)

# View the draws for lambda for states 1 and 5.
plot(log(res[[1]][,’lambda[1]’]), main=expression(Samples~of~Lambda[1]))
plot(log(res[[1]][,’lambda[5]’]), main=expression(Samples~of~Lambda[5]))

# Get the predicted ILI trajectory for national level.
mat_disp = matrix(NA,nrow=dat$NT,ncol=3)
for(t in 1:(dat$NT)){
  tmp = 100*res[[1]][,paste0(’futurey_nat[’,t,’]’)]
  mat_disp[t,2] = mean(tmp)
```

```
  mat_disp[t,c(1,3)] = quantile(tmp, c(0.025,0.975))
}

# Plot observed ILI, then overlay model predictions for the rest of the season.
plot( 1:(dat$NT), c(mat_disp[1:(dat$nobs),2],rep(NA,dat$NT-dat$nobs)), ylim=range(c(0,mat_disp)),
      xlab='Epi time', ylab='ILI')
inds=(dat$nobs+1):(dat$NT); lines(inds,mat_disp[inds,2]) # Predicted mean ILI.
lines(inds,mat_disp[inds,1], lty=2); lines(inds,mat_disp[inds,3], lty=2) # Predicted 95% credible interval.
```

Supplementary Note 2.4. Instructions for use

Users who wish to use Dante on their own data should create an `R` list containing the following named variables: `NR_state`, `NR_region`, `NS`, `NT`, `y_state`, `yobs_region`, `yobs_nat`, `census_weights`, `fcstseason`, and `nobs`. Descriptions of each variable may be found in Supplementary Note 2.1. Note that the ordering between elements in the list entries must be consistent (e.g., the order of states by row in `dat_state` and `census_weights` must match).

Following data creation, assuming `Dante.bug` (i.e., the code in Supplementary Note 2.1) is in the current working directory and the data are named `dat`, the model can be run by simply running `jags.model(file="Dante.bug", data=dat)` in `R`.

Supplementary Note 2.5. Attribution

If you use the Dante in your research work, please cite the main paper.

A minimal working code example can be found at `https://github.com/lanl/dante` and is open source under the BSD-3 License. Redistribution and use in source and binary forms, with or without modification, are permitted provided that the following conditions are met:

1. Redistributions of source code must retain the above copyright notice, this list of conditions and the following disclaimer.

2. Redistributions in binary form must reproduce the above copyright notice, this list of conditions and the following disclaimer in the documentation and/or other materials provided with the distribution.

3. Neither the name of the copyright holder nor the names of its contributors may be used to endorse or promote products derived from this software without specific prior written permission.

Supplementary Note 3. Details for computing average standardized week-to-week (w)ILI volatility

We developed a measure called "average standardized week-to-week volatility," (v), to measure how volatile or smooth (w)ILI is for region r. The average standardized week-to-week volatility, displayed in Figure 3, is computed as follows:

$$v_r = S^{-1} \sum_{s=1}^{S} v_{rs}, \tag{34}$$

where

$$v_{rs} = \sqrt{(T-1)^{-1} \sum_{t=2}^{T} (y^*_{rst} - y^*_{rs,t-1})^2}. \tag{35}$$

Furthermore,

$$y^*_{rst} = (y_{rst} - \bar{y}_{rs})/\sigma_{rs}, \tag{36}$$

where

$$\bar{y}_{rs} = T^{-1} \sum_{t=1}^{T} y_{rst}, \tag{37}$$

$$\sigma_{rs} = (T-1)^{-1} \sum_{t=1}^{T} (y_{rst} - \bar{y}_{rs})^2, \tag{38}$$

S is the number of flu seasons, and T is the number of epidemic weeks. y^*_{rst} is the region/season standardized (w)ILI which has a mean of zero and a standard deviation of one by construction. Without standardizing, regions with higher (w)ILI would appear to be more volatile simply because they have higher levels of (w)ILI. The average standardized week-to-week volatility for state r represents a measure of how volatile the ILI time series is for that state.

Supplementary Note 4. State ILI

Supplementary Figure 3 displays ILI for all states for the 2010 through the 2017 flu seasons. The black line represents the average national wILI over that same time period for reference. Significant state-to-state variability exists, with some states having ILI consistently above the national average (e.g., Alabama, Oklahoma, Puerto Rico, Texas), and some states consistently below the national average (e.g., Montana, New Hampshire, Oregon, Rhode Island). Some states are more volatile (e.g., North Dakota, Puerto Rico) while some are less volatile (e.g., Virginia, New York City).

Supplementary Note 5. Census weighted ILI to wILI

Information about HHS Regions is available at `www.hhs.gov/about/agencies/iea/regional-offices/index.html`. Supplementary Figure 6 shows wILI as reported by the CDC on the x-axis against the 2010 US Census weighted constructed wILI, calculated as a weighted average of state-level ILI with weights proportional to 2010 US Census population counts on the y-axis. Very close agreement is shown, indicating that wILI can be computed as a weighted combination of state-level ILI.

Supplementary Note 5.1. Census weighted ILI to wILI example

For concreteness, we show that wILI on EW49 of 2017 for HHS Region 9, reported as **2.596**, can be computed as the weighted combination of state ILI. HHS Region 9 has four states: Arizona, California, Hawaii, and Nevada. The 2010 US Census population estimates for the four HHS Region 9 states are 6,407,774 (AZ), 37,320,903 (CA), 1,363,963 (HI), and 2,702,464 (NV), for a total of 47,795,104 people in HHS Region 9. The state weights are then 0.134 (AZ), 0.781 (CA), 0.029 (HI), and 0.057 (NV). CDC reported ILI for EW49 of 2017 for those states are 3.284 (AZ), 2.498 (CA), 4.341 (HI), and 1.434 (NV).

The state-weighted wILI estimate for HHS Region 9 is:

$$
\begin{gathered}
w_{AZ} * ILI_{AZ} + w_{CA} * ILI_{CA} + w_{HI} * ILI_{HI} + w_{NV} * ILI_{NV} = \\
0.134 * 3.284 + 0.781 * 2.498 + 0.029 * 4.341 + 0.057 + 1.434 = \mathbf{2.596},
\end{gathered}
$$

the same wILI value reported by the CDC.

Supplementary Note 6. Forecasting targets

The forecasting targets as selected by the CDC for the FluSight challenge are defined as follows:

- Short-term forecasts ($n-$week ahead):
  - The (w)ILI percentage for each target week n weeks ahead of the most recently released (w)ILI data, where $n = 1, \ldots, 4$.
- Peak intensity:
  - The highest value of (w)ILI in a given season.
- Peak timing:
  - The week(s) in which the highest value of (w)ILI in a given season is reached.
- Onset:
  - The first week in which (w)ILI reaches or exceeds the given regional/national baseline value for three consecutive weeks in a season. Note that 'baseline,' a threshold used to define epidemic onset, is calculated by adding two standard deviations to the mean observed ILI levels in non-flu weeks during the previous three influenza seasons; see `https://www.cdc.gov/flu/weekly/overview.htm`.

Due to backfill, the CDC sets a validation date at which to record all of the above 'validation' values for each forecast challenge. For this paper, data for all seasons were collected from the CDC's website on Friday, October 12th of 2018 and validation %ILI values are considered those present in the collected data set.

The ILINet national and regional baseline values are available at `www.cdc.gov/flu/weekly/overview.htm`. Note that state data do not have defined historic baseline values.

A visual example of Dante's national-level predictions for each of the forecasting targets may be found in Figure 7.

Supplementary Note 7. Forecasting example

Supplementary Figure 2 shows Dante's forecasts submitted to the FluSight challenge for the 2018/19 flu season for HHS region 6 and all states within. Each column represents a date the forecast was made. State forecasts are produced via the state-level model of Dante, while the

HHS region 6 forecasts are produced via the aggregation model of Dante. Supplementary Figure 2 illustrates how correlated errors propagate in the aggregation process. All state-level forecasts made on “2019-01-13” underestimate the impending increase in ILI to varying degrees. Because all states miss in the same direction, that miss propagates through the aggregation process to the HHS region 6 forecast, which also underestimates the impending increase in ILI. Thus, though the forecasts are coherent across scales, they necessarily inherit the benefits and challenges of correlated errors.

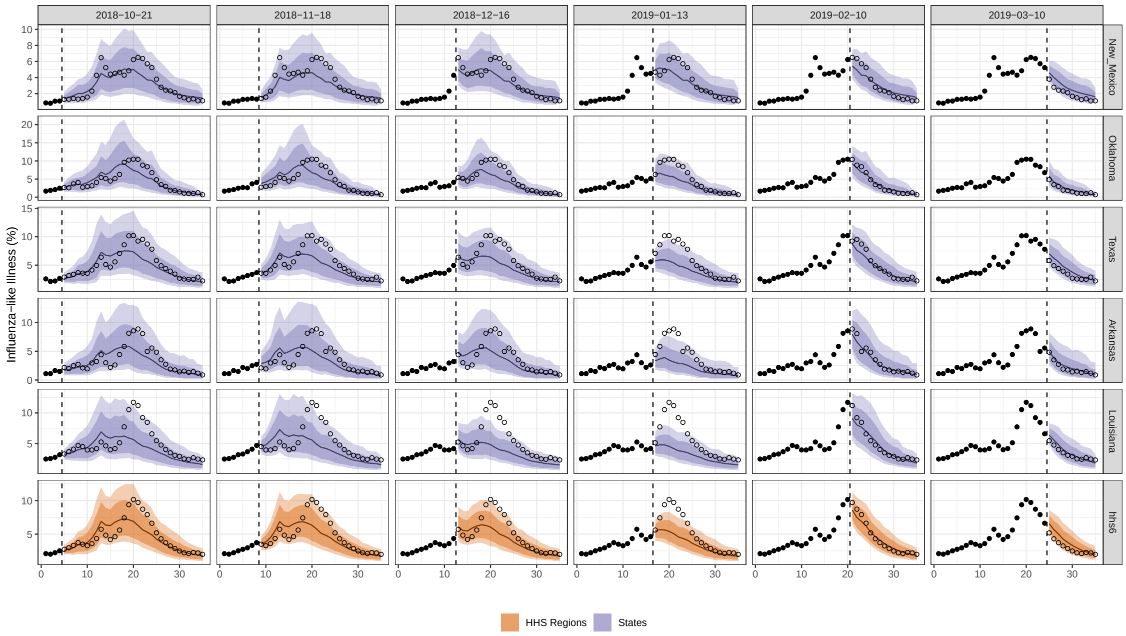

Supplementary Figure 2: HHS region 6 forecasts for the 2018/19 season made at different dates throughout the flu season (columns) for all states in HHS region 6 (first five rows) and the aggregated forecasts for the region (last row). Black points to the left of the dashed vertical line are observed values; open points to the right are unobserved. Ribbons represent 80% and 95% prediction intervals.

Supplementary Note 8. Scoring procedure

Scoring protocols used in this paper correspond to the stated scoring procedure used by the CDC’s FluSight challenge for the 2018/19 season. Complete national and regional FluSight challenge description and scoring guidelines may be found by navigating to the ‘Guidance Documents’ page from `predict.cdc.gov/post/5ba1504e5619f003acb7e18f`. State FluSight guidelines are available by navigating to the ‘Guidance Documents’ page from `https://predict.cdc.gov/post/5ba5389fa983f303b832726b`.

All forecasts are evaluated using the validation %ILI data. That is, the larger effects of backfill

should have subsided and the ILINet observations used are stable from week-to-week. This lack of backfill leads to a relatively higher performance of both Dante and DBM relative to their performance in a real-time FluSight challenge.

Supplementary Note 8.1. Scoring by target

Note that for the purposes of finding the peak timing, onset, and scoring, all (w)ILI values are rounded to the nearest tenth. For example, a reported (w)ILI of 5.387% would become 5.4%. The scoring bin width is one tenth of a percentage for short-term targets and peak intensity, and one week for peak timing and season onset.

- Short-term forecasts ($n-$week ahead):
  - The probability assigned to the correct bin plus the probabilities assigned to the five preceding and proceeding bins are summed.
  - For example, if (w)ILI at week 49 is 2.5%, the probabilities assigned to all bins ranging from 2.0% to 3.0% inclusively are summed in the 1-week-ahead forecast from week 48 to get that week's 1-week ahead forecast skill.
- Peak intensity:
  - The probability assigned to the correct bin plus the probabilities assigned to the five preceding and proceeding bins are summed.
  - For example, if (w)ILI peaks at 5.4%, the probabilities assigned to all bins ranging from 4.9% to 5.9% inclusively are summed to get the skill for peak timing.
- Peak timing:
  - The probability assigned to the correct week(s) plus the probabilities assigned to the immediately preceding and proceeding weeks are summed.
  - In the case of multiple peaks, the probability assigned to each correct week plus the probability assigned to the non-overlapping preceding and proceeding weeks for each peak is summed; each week's probability is only counted once.
  - For example, if (w)ILI peaks at 5.4% on weeks 3 and 5, then the probabilities assigned to weeks 2, 3, 4, 5, and 6 are summed to get the skill for peak timing.
- Onset:
  - The probability assigned to the correct week(s) plus the probability assigned to the immediately preceding and proceeding weeks is summed.

- For example, if the onset is week 48, then the probabilities assigned to weeks 47, 48, and 49 are summed to get the skill for onset.
- Note that onset is not defined at the state level.

For all targets, if the correct week/bin is near the first or last possible bin then the total number of weeks/bins summed for scoring is reduced accordingly. For example, if the validation (w)ILI value is 0.3%, then only bins ranging from 0% to 0.8% will be counted for scoring $n-$week ahead predictions (there won't be extra bins counted above 0.8% to 'make up for' the decrease in the number of lower bins).

Supplementary Note 8.2. Averaging scores

In this paper, forecasts are made beginning at epi-time 5 (i.e., epi-week 45, after four weeks of data from a given season are available) and the last forecast is made at epi-time 29 (i.e., after the influenza season has long been over).

When calculating an overall score for a given location, season, and/or model, we take the geometric mean of a subset of the scores for that location/season/model. That is, relevant log skills across the targets of interest are averaged and the result is exponentiated. However, the weeks at which a forecast is made and the target chosen impact whether or not a given week's forecast skill is included. The scoring inclusion window used in this paper is the intersection of this paper's forecast window (epi-time 5 through 29) and the CDC definition for weeks to score (discussed below). Note that when skill is 0 (i.e., when the probability assigned to the scorable bin(s) by the model is 0), the log score is set to -10 for that target.

Supplementary Note 8.2.1. CDC's evaluation period

For state forecasts, all weeks are included in the average score calculation because there are no state-level baseline values. For regional and national forecasts, the CDC chooses evaluation periods based on utility of said forecasts. For all seasonal targets (i.e. onset, peak timing, and peak intensity), the CDC evaluation period begins with the first forecast submission. For onset, the CDC evaluation period ends six weeks after the validation onset. For peak timing and intensity, the evaluation period ends after (w)ILI is observed to go below baseline for the final time during an influenza season (we interpret this to mean the scored weeks are those up to and including the first week below baseline after which all weeks are below baseline). For short-term forecasts (i.e., $n-$week ahead predictions) the CDC evaluation period begins four weeks prior to the observed onset week and ends three weeks after (w)ILI is observed to go below baseline for the final time

during an influenza season. We assume all windows are inclusive (i.e., three weeks after implies that the third week out *is* counted). The idea is that forecasts within these windows are those that would be most practically useful.

Supplementary Note 8.2.2. Averaging scores example

As an example, suppose the goal is to generate an overall score for Dante's onset forecasts for HHS6 in 2014. The historical baseline for HHS6 in 2014 was 3.2, and the observed onset week was week 47 (epi-time 8). The CDC evaluation period would include forecasts made during epi-time 1 through 14, inclusive. We produce model forecasts during epi-time 5 through 29. The overall score would be the geometric mean (i.e., the exponentiated log average score) for HHS6 onset forecasts made on epi-time 5 through 14, inclusive, in 2014. For the purposes of illustration, we round the following values. Specifically, the skill at epi-time 5 (week 44) is 0.27, meaning that in week 44 the sum of the probability assigned to weeks 46, 47, and 48 (i.e., the validation onset week and its preceding and proceeding week) was 0.27. At epi-time 6, the skill is 0.22. At epi-times 7 through 14 the skills are 0.10, 0.68, 0.99, 0.99, 0.99, 0.99, 0.99, and 0.99. Thus the summary score for onset of the 2014/2015 season of flu in HHS6 is:

$$e^{\frac{\ln(0.27)+\ln(0.22)+\ln(0.10)+\ln(0.68)+\ln(0.99)+\ln(0.99)+\ln(0.99)+\ln(0.99)+\ln(0.99)+\ln(0.99)}{10}} = 0.57$$

In order to get an overall score for Dante's forecasts for HHS6 across all targets and seasons, the geometric mean would be taken of all of the relevant scores from each season-target combination.

Supplementary Note 8.3. Proper scoring

Rather than considering bins around the truth (e.g., as in short-term forecasts when the probability assigned to the correct bin plus the probabilities assigned to the five preceding and proceeding bins are summed), the proper score reported in the main text only counts probabilities assigned to the correct bin. Averaging is performed analogously to the improper CDC scoring.

Supplementary Note 9. Highest posterior density interval width calculation

This section provides details for the highest posterior density (HPD) interval width calculations corresponding to Figure 6. The HPD interval widths are computed from the binned submission files, following the format of the FluSight challenge (described in Supplementary Note 8). In the submission files for all targets, a probability is assigned to each bin. We order the bins according to the probability assigned to it, starting with the bin with the largest assigned probability. Then, to compute the $X\%$ HPD interval width, we add up the probability of the ordered bins until the running sum exceeds $X/100$ for the first time. The smallest number of bins required for the running probability sum to exceed $X/100$ is multiplied by 0.1% (the bin width), resulting in the estimated HPD interval width in units of the short-term target. For concreteness, if 0.8 and 0.15 probability were the two largest probabilities assigned to bins, respectively, then the 90% HPD interval width would be computed as 0.2%.

We note that there is one bin from 13% to 100% with a width not of 0.1% but of 87%. We treat all bins as having the width of 0.1%, an admitted underestimation of HPD interval widths. We note, for comparison purposes, the same underestimation is done for both Dante and DBM.

Supplementary Note 10. Data cleaning

The state data were pulled from the CDC's website on Friday, October 12th of 2018 before being cleaned. In the data, the `ili` variable is the percent of patients having ILI symptoms out of the total number of patients seen. For the majority of observations, the `ili` variable is equivalent to 100 times the `ilitotal` variable divided by the `total_patients` variable. In the original data set when `total_patients`$= 0$, `ili`$= 0$. We clean the data by setting `ili` to `NA` when `total_patients`$= 0$.

ili	ilitotal	total_patients
0.586	3	512
0.242	1	413
0	0	0

ili	ilitotal	total_patients
0.586	3	512
0.242	1	413
NA	0	0

Supplementary Table 1: (Left) The relationship between `ili`, `ilitotal`, and `total_patients` pre-cleaning. Note that in the third row `ili` is 0 when `total_patients` is 0. (Right) The relationship between `ili`, `ilitotal`, and `total_patients` post-cleaning. In the third row the 0 is replaced with an `NA`.

Note that in recent releases of (w)ILI data, the presence/absence of `NA` coding may differ from that used in older data releases. Should this model be used for new data, cleaning said data with the logic used above for when (w)ILI should be 0 vs. `NA` is recommended.

Supplementary Note 10.1. Missing state-level data

After using the above data cleaning techniques, 3 missing state-season-week observations persisted (see Supplementary Figure 8), excluding completely missing state-seasons (e.g., Florida, for which no data were available for any state-season-week, and Puerto Rico in 2012). For a given state-season, if any weeks are missing data it is theoretically possible for that week to be the validation peak week. Thus, the seasonal targets of peak timing and peak intensity have `NA` for a ground truth in instances in which that state-season has any missing data and the forecast skill for those seasonal targets must also be `NA`.

Practically speaking, however, not every missing week need preclude scoring seasonal targets for a whole state-season. For example, it is incredibly unlikely for influenza to peak in mid-May. On the other hand, a missing week in mid-December right near the highest observed ILI value has a much higher chance of being the validation peak. For determining which weeks should be considered 'irrelevant' missing weeks, we calculate the historic minimum and maximum observed peak timing from the 2010/2011 season to the 2017/2018 season for each state. We then utilize this state-specific min/max historic peak timing minus/plus a three week 'buffer' as the (inclusive) window in which an `NA` will lead to that state-season seasonal forecasts are determined to be unscorable.

Supplementary Note 10.2. Missing region-level data

There are no `NA`s present in the region-level data. When a state-season-week is missing for a given region, that state is omitted from the weighted average calculation for that region's wILI and the weights are adjusted accordingly.

Supplementary Note 10.3. Model output cleaning

As mentioned previously, a skill of 0 means that the log-score is set to -10 for that target per CDC guidelines. In practice, we place a small probability in every bin, to eliminate the possibility of incredibly damaging -10s. For peak week and onset, we choose 0.00018 to be the smallest probability in every bin, where the worst possible log score will then be $\ln(3 * .00018) \approx -7.5$ (using the multibin log score); for peak percentage and short-term forecasts, we choose .00005

so the worst possible log score will be about $\ln(11 * .00005) \approx -7.5$. Note that these particular padding values were chosen as a simple risk mitigation step. Further study could be done to determine some "optimal" pad for scoring, but for the purposes of this paper the main goal was comparability of Dante and DBM so the same numeric pad was used for each.

Supplementary Note 11. Forecast skill

We consider detailed results for Danta's forecast skill for each prediction time broken down by target-season at the national level and the state level for two example states. Discussion is based on Supplementary Figures 9–12. Note that in Supplementary Figures 9–11 the x-axis shows the time at which the forecast was made, while the (w)ILI data that are overlaid have been shifted to the left n weeks for each n-week ahead skill plot. For example, for the 1-week-ahead forecast skill plots the skill plotted at epi time 5 is the skill in forecasting (w)ILI at epi time 6, while the scaled inverse (w)ILI plotted at epi time 5 is the corresponding (w)ILI value from epi time 6.

Supplementary Note 11.1. National skill

Supplementary Figure 9 shows Dante's forecast skill for each prediction time broken down by target-season. Early in the season, the seasonal targets each tend to have low scores; by the end of the season (after the seasonal metric has been observed) the scores increase to near 1. The short-term forecasts are much more variable, but broadly skill is inversely related to wILI.

Consider the first row of Supplementary Figure 9. By at most three weeks after an onset has been observed (the defining feature of onset being the consistent wILI above baseline for three weeks), the onset skill reaches near 1 because the model uses the observed wILI data as its prediction upon observation and there is no backfill to contend with, unlike with current-season forecasts. Of note is that for 2015, which was an unusually late-peaking season, the onset skill is near 0 early in the season because at this point the model is basing its predictions on previous seasons' trajectories, meaning that a low-flu late-peaking season has a low probability of occurring before observing much data from the current season.

The final four rows of Supplementary Figure 9 illustrate the inverse relationship between skill and wILI. This general phenomenon is likely partly due to the nature of the scoring for short-term targets. Recall that the probabilities assigned to the bins from -0.5 to 0.5 away from the validation wILI level are summed. If the validation wILI level is very high, the model has more room to be 'wrong' even if it is generally right that levels are high. For example, if the validation wILI one week ahead is 7 and the model puts most of its weight around a wILI of 6, the model will score

very poorly even though the concept of high wILI is correct. On the other hand, if the validation wILI is low and the model predicts that wILI is low, more probability will tend to be near the truth simply because there are fewer 'low' wILI values. The higher skill during early/late season is likely also partly due to the fact that wILI behaves much more consistently at the beginning/end of seasons when flu is not circulating as heavily, and week-to-week changes tend to be smaller and occur more gradually, so the trajectory learned from previous seasons and other states in the model is more useful to a new given state-season combination at the tails of the season.

Supplementary Note 11.2. State skill

Supplementary Figures 10 and 11 show target-season skill plots for two example states (Alabama and Montana, respectively). The same general trends with seasonal target skill for national data are found in the state-level seasonal target skills with some notable differences. For many target-season combinations, the rise of state-level peak incidence/timing (pi/pt) skill to near 1 occurs later after the peak has occurred than for the national pi/pt skill. For example consider the pi/pt skill for Montana in the 2016 season (the first and second rows in the second from the rightmost column in Supplementary Figure 11). After the peak has occurred, it is nearly the end of the season before pi/pt skill rises to near 1. Looking at the bottom row in the second from the rightmost column in Supplementary Figure 12, which shows the validation ILI for Montana in 2016, it is evident that this was a particularly early/weak-peaking and low-magnitude season for Montana. The model had trouble telling that a peak had in fact occurred, so some probability remained allocated to later peak timing and higher peak incidence. In Montana, pi/pt skill rises almost immediately as the peak occurs in 2015, likely because this is a fairly prominent (by Montana standards) peak occurring late in the season, so the model very readily places high probability on it being the peak after its occurrence.

The skill for short-term targets is in general much higher for Montana than for Alabama, particularly for seasons in which ILI levels in Montana lack much of a distinct peak (i.e., 2016 and 2017). The inverse relationship between ILI and and short-term forecast skill is less consistent at the state level than it was at the national level. In Alabama (the bottom four rows of Supplementary Figure 10), the short-term forecasts in 2015 and 2016 track extremely well with inverse ILI; in particular, note the spiked pattern in both skill and inverse ILI from epidemic time (ET) 11 to 15 for 4-week ahead forecasts in 2016 (shifted right by one with each decrease in week, so 12 to 16 for 3-week ahead, etc.). On the other hand, the 1-, 2-, and 4-week ahead forecasts in 2014 corresponding to the peak week (made at ET 12, 11, and 9, respectively) have high skill and correspond to high ILI. When looking at the corresponding subplot in Supplementary Figure 12, we see that the slope to the peak is relatively constant save for the 3rd week before the peak

for which the 3-week ahead forecast was poor, suggesting that the high performance may be due to the consistency of the observed path at the time of forecast with the future path. Another interesting feature of forecasts in Alabama is that short-term skill tends not to begin as high as that of national forecasts. Of note, consider the near-0 starting skill at ET 5 in 2012 for 3- and 4-week forecasts. Looking at the middle row of the leftmost column of Supplementary Figure 12, we see that in 2012 ILI in Alabama experienced a dip at ET 4, followed by a small rise in ET 5 and increasingly steeper rises over the subsequent 4 weeks. The 3/4-week forecast from ET 5 is unable to anticipate this rise, hence the extremely poor skill.

Supplementary Note 12. Accuracy comparison

Accuracy is measured by mean squared error (MSE) of model point predictions. In the main paper, we showed that Dante was both more skillful (i.e., higher forecast skill scores) and more confident (i.e., narrower 90% HPD interval widths) than DBM. We also showed that Dante outperformed DBM in terms of accuracy across all geographic scales. However, we did now show the relative performance of Dante and DBM broken down by region, season, or target. Supplementary Figures 13 and 14 provide this additional information. Note that these plots are analogous to the skill plots in the main paper, but compare MSE rather than skill. Averages were calculated using the same set of weeks as defined by the CDC guidelines for scoring forecast skill.

Specifically, Supplementary Figure 13 shows the difference in accuracy between Dante and DBM for each state, region, and nationally. Dante outperformed DBM for the majority of geographic regions, but its relative performance is less consistently better as measured by accuracy than it is by skill.

Supplementary Figure 14 shows accuracy broken down by targets (left) and flu seasons (right) for each geographic scale. Dante outperformed DBM for all scales and targets, except for onset nationally and for peak intensity (PI) regionally and by state. Relative magnitude of differences for peak timing (PT) appear higher because the MSE are in terms of the target (i.e., MSE for PT is in terms of squared weeks). Dante also outperformed DBM for the majority of scales and flu seasons, except for 2013 nationally and for 2014 and 2016 by state.

Supplementary Note 13. Probability densities

Supplementary Note 13.1. Normal(μ, σ^2)

The probability density function for the Normal distribution is

$$f(x|\mu,\sigma^2) = \frac{1}{2\pi\sigma^2}\exp\Bigg(-\frac{(x-\mu)^2}{2\sigma^2}\Bigg) \tag{39}$$

where $x \in (-\infty,\infty)$, $\mu \in (-\infty,\infty)$, and $\sigma^2 > 0$. The mean and variance for a Normal distribution are $\mathrm{E}(X) = \mu$ and $\mathrm{Var}(X) = \sigma^2$.

Supplementary Note 13.2. t(μ,λ, k)

The probability density function for the non-standardized t-distribution is

$$f(x|\mu,\lambda,k) = \frac{\Gamma(\frac{k+1}{2})}{\Gamma(\frac{k}{2})}\Bigg(\frac{\lambda}{k\pi}\Bigg)^{\frac{1}{2}}\Bigg\{1+\frac{\lambda(x-\mu)^2}{k}\Bigg\}^{-\frac{(k+1)}{2}} \tag{40}$$

where $x \in (-\infty,\infty)$, the non-centrality parameter $\mu \in (-\infty,\infty)$, the precision $\lambda > 0$, and the degrees of freedom $k > 0$. For $k > 1$, $\mathrm{E}(X) = \mu$. For $k > 2$, $\mathrm{Var}(X) = \frac{k}{\lambda(k-2)}$.

Supplementary Note 13.3. Gamma(α, β)

The probability density function for the Gamma distribution is

$$f(x|\alpha,\beta) = \frac{\beta^\alpha}{\Gamma(\alpha)}x^{\alpha-1}\exp(-\beta x) \tag{41}$$

where $x \in (0,\infty)$, $\alpha > 0$ is the shape parameter and $\beta > 0$ is the rate parameter. The mean and variance for the Gamma distribution are $\mathrm{E}(X) = \frac{\alpha}{\beta}$ and $\mathrm{Var}(X) = \frac{\alpha}{\beta^2}$.

Supplementary Note 13.4. Beta(α, β)

The probability density function for the Beta distribution is

$$f(x|\alpha,\beta) = \frac{\Gamma(\alpha+\beta)}{\Gamma(\alpha)\Gamma(\beta)}x^{\alpha-1}(1-x)^{\beta-1} \tag{42}$$

where $x \in [0, 1]$, $\alpha > 0$, and $\beta > 0$. The mean and variance for the Beta distribution are $\mathrm{E}(X) = \frac{\alpha}{\alpha+\beta}$ and $\mathrm{Var}(X) = \frac{\alpha\beta}{(\alpha+\beta)^2(\alpha+\beta+1)}$.

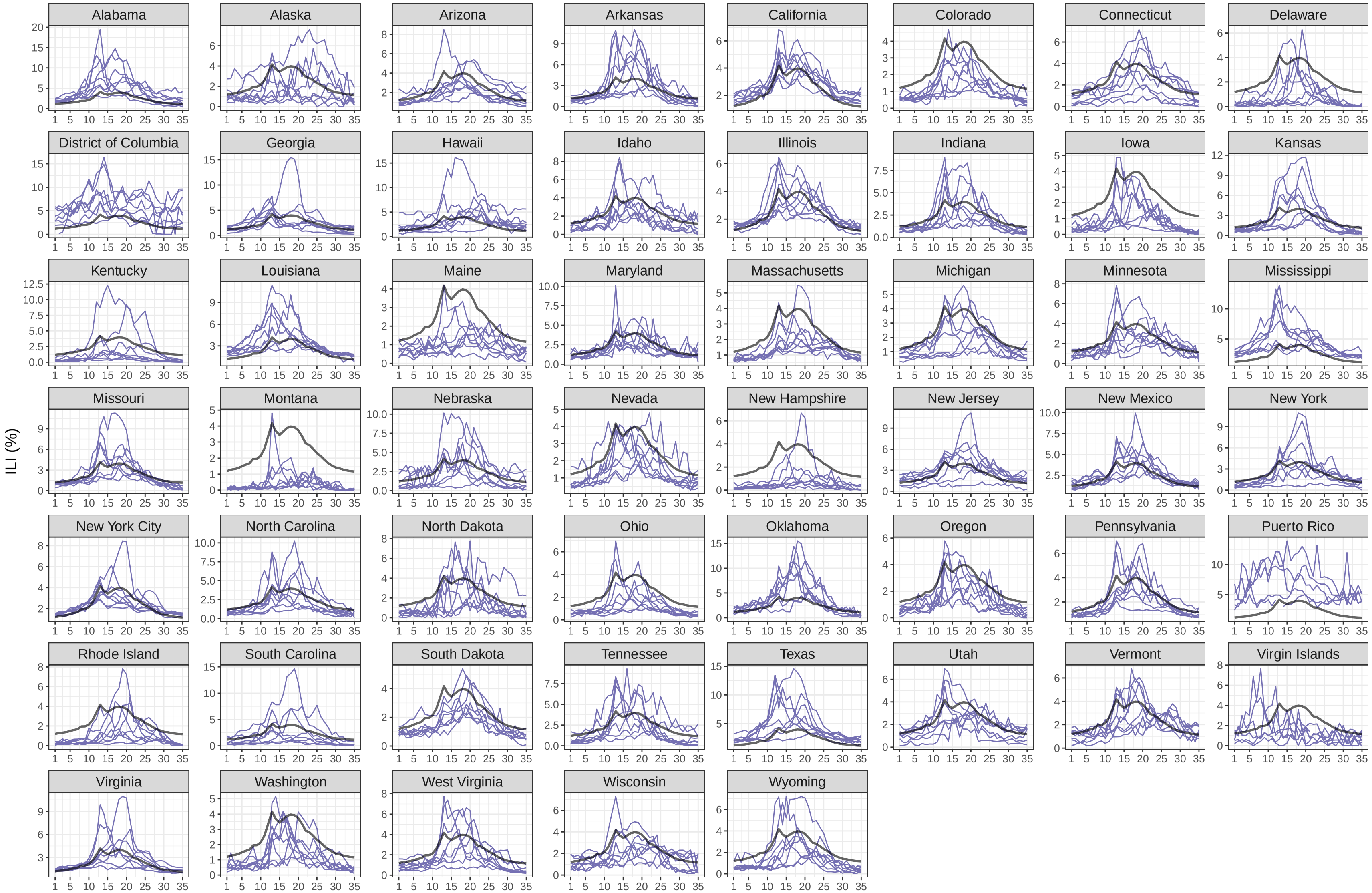

Supplementary Figure 3: ILI for the 2010 through 2017 flu seasons (grey lines) for all states. Black line is the average wILI nationally for the same time period. The weeks of the flu season (x-axis) run from roughly the first week of October (x=1) through the end of May (x=35).

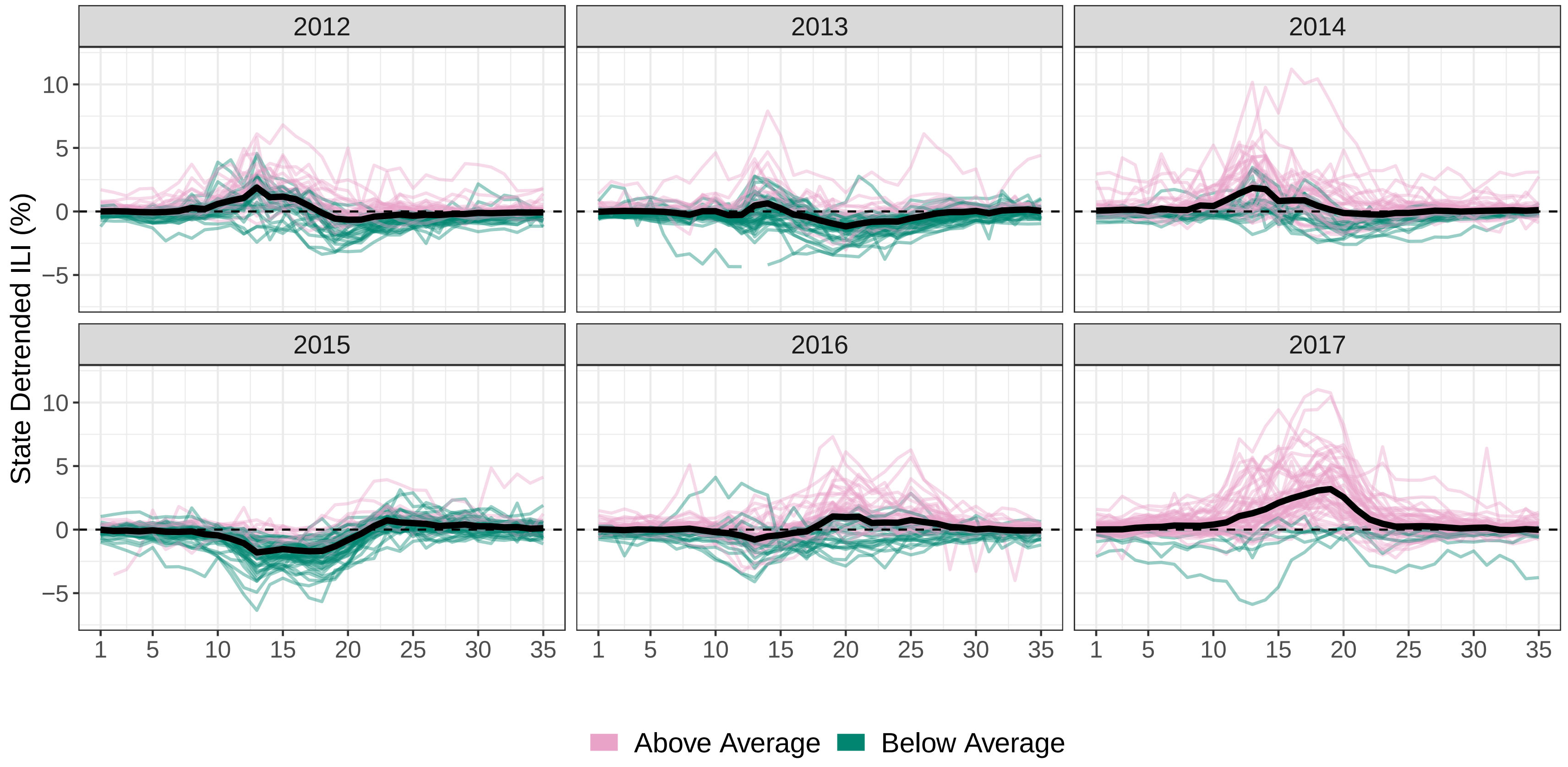

Supplementary Figure 4: State detrended ILI time series where the state detrended time series is ILI for a given season minus the average ILI for the state averaged over all seasons. Pink/dark green lines correspond to seasons where ILI for that season was above/below its state-specific average, respectively. The black line for each season is the average state detrended ILI trajectory, averaged over all states within a season. 2015 was a mild season for the majority of states, while 2017 was an intense season for the majority of states.

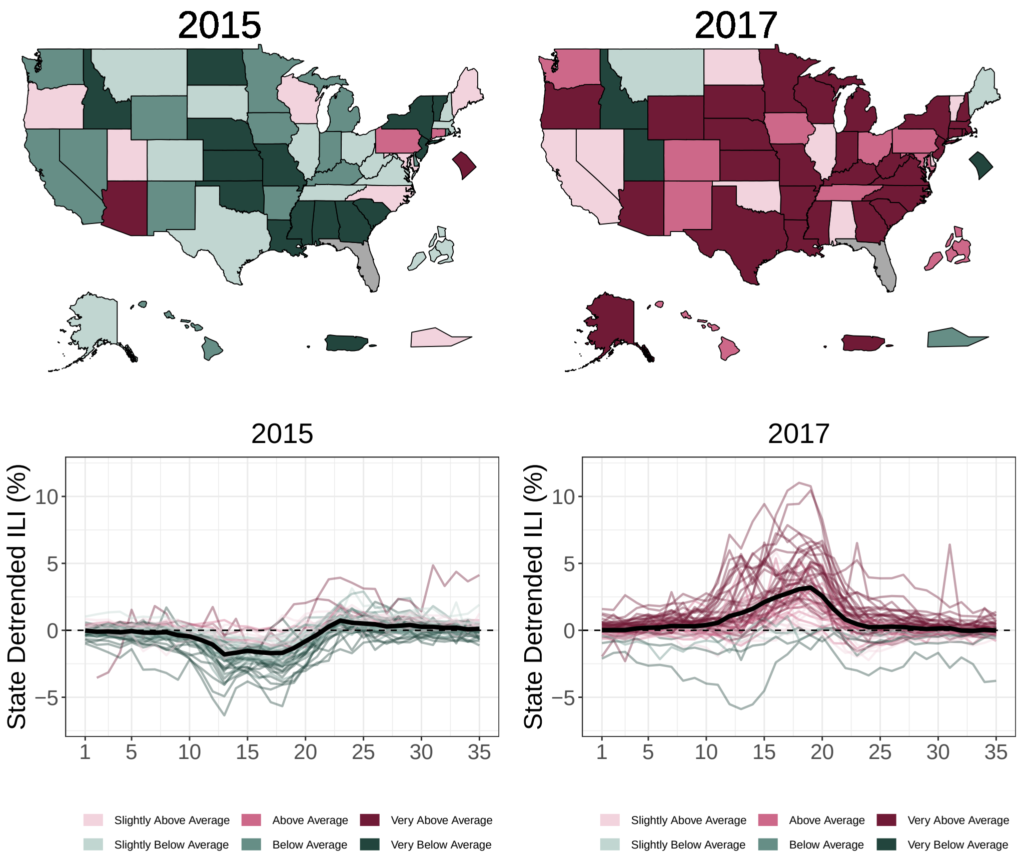

Supplementary Figure 5: (Top) Green states denote states with ILI less than their state-specific averages while pink states are states with ILI above their state-specific averages. Intensity of color indicates the magnitude above or below its average a given state was during that season. 2015 was a mild flu season for the majority of states relative to their state-specific average ILI, while 2017 was an intense flu season for the majority of states, indicating that season-to-season effects can affect most of the country. Data unavailable for Florida. States displayed outside of the contiguous US are geographically not to scale. (Bottom) State detrended ILI for the 2015 and 2017 flu seasons, where state detrended ILI is ILI for a state/season minus ILI for that state averaged over all seasons. Positive/negative state detrended ILI means ILI for that season was above/below the state-specific average, respectively.

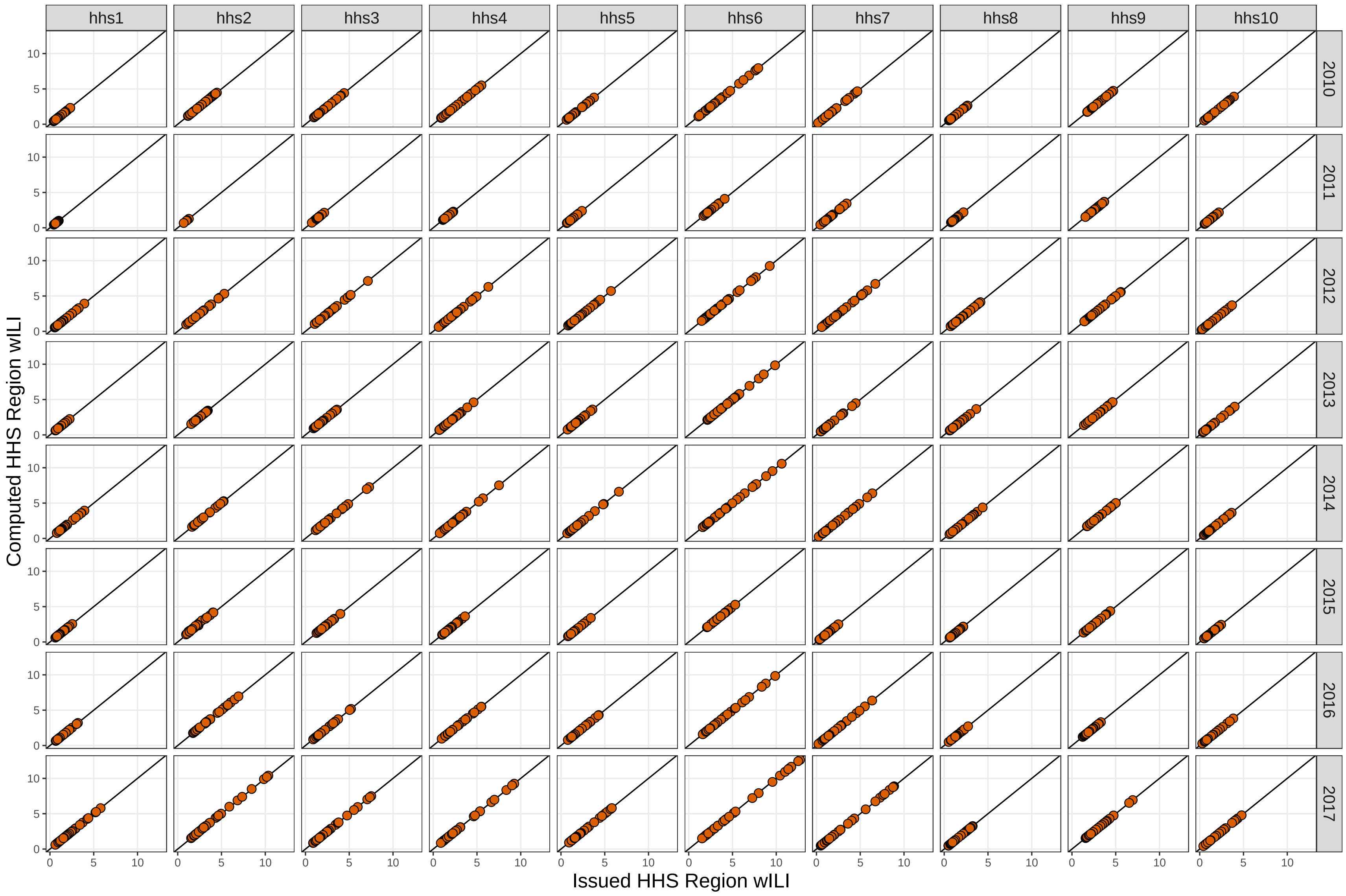

Supplementary Figure 6: Health and Human Services (HHS) Region weighted influenza-like illness (wILI) issued by the Centers for Disease Control and Prevention (CDC) on the x-axis against HHS Region wILI computed as the weighted combination of state-level ILI issued by the CDC on the y-axis. The weights are proportional to the 2010 US Census population estimates. Computed and issued HHS Regional wILI are nearly identical. Each panel corresponds to an HHS Region (column) and a flu season (row).

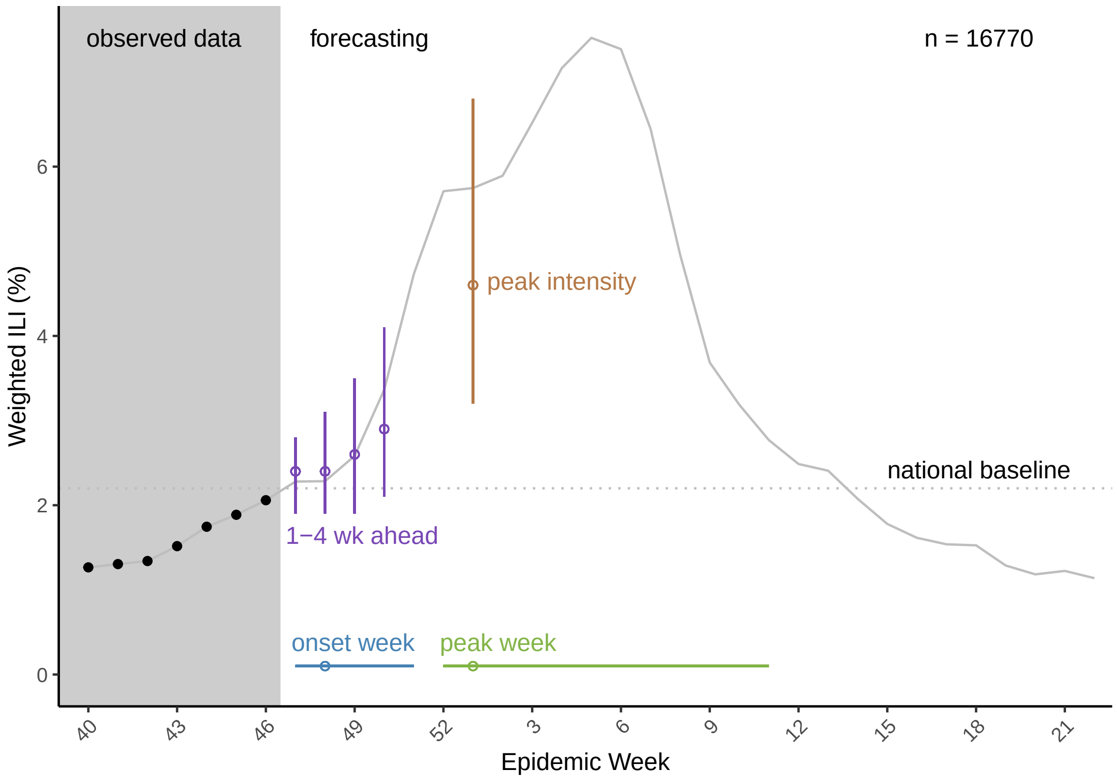

Supplementary Figure 7: Top: national wILI for the 2012 through 2017 flu seasons, i.e. those used in the leave-one-season-out training and testing of DBM and Dante. The 2017 flu season, the hold-out season considered in the lower plot, is emphasized in black. Bottom: Dante's national forecasts for the CDC targets in 2017 made with (w)ILI data from epidemic week 46 being the last available data. Posterior mode and 95% HPD interval for each target are shown as an open point and line, respectively. For these forecasts, the model is utilizing all data from the 2012 through 2016 flu seasons, and the first 7 weeks of data from the 2017 flu season (indicated by the solid black points); the sample size in terms of state-week-season observations is shown in the top right. The remainder of the 2017 flu season (shown in the bottom figure as a grey line) has yet to be observed.

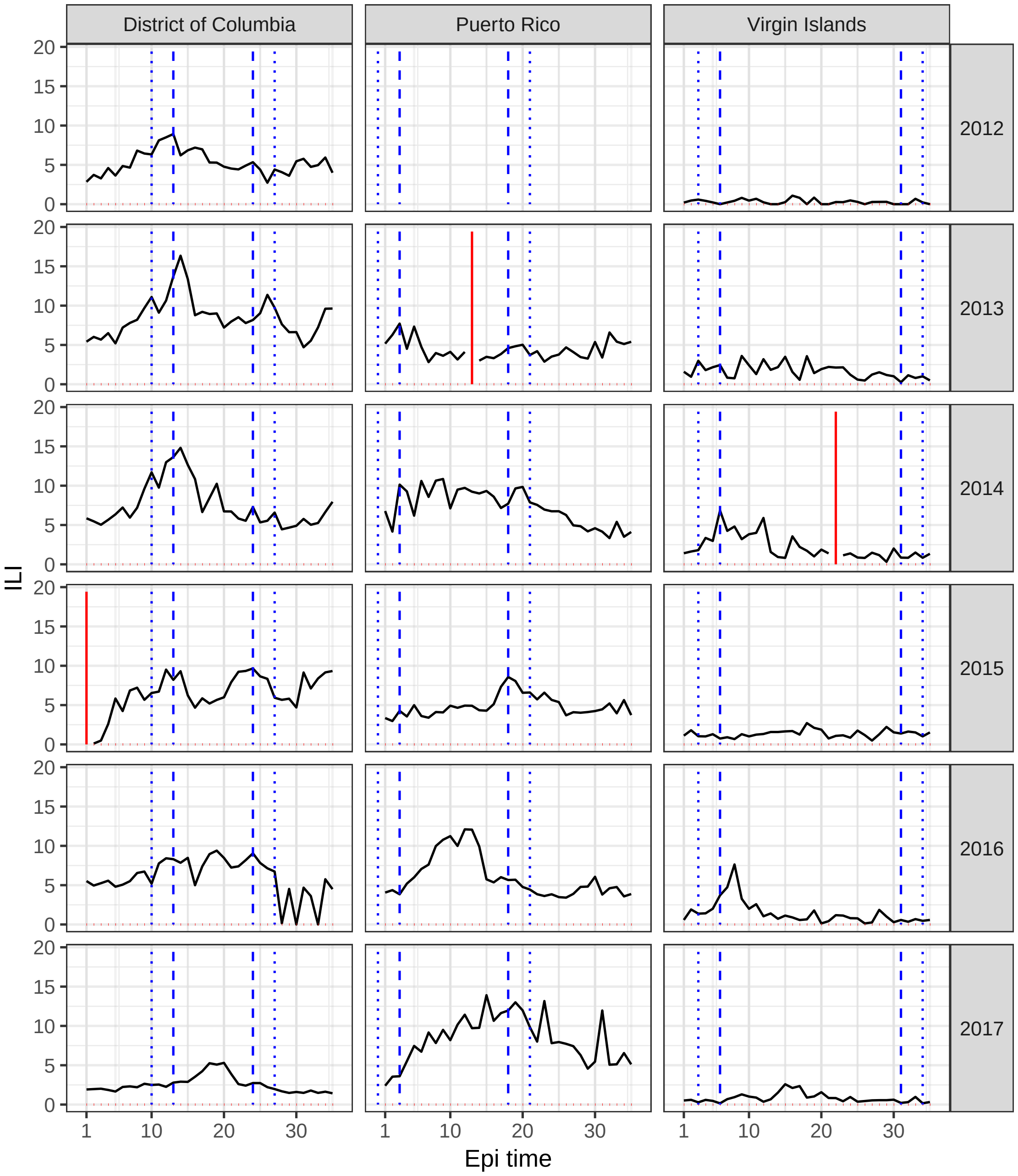

Supplementary Figure 8: The three missing observations among state-season combinations which are not completely unobserved are denoted by the red vertical lines in each subplot. The inner long dashed blue lines show the minimum and maximum historic peak weeks from the 2010/2011 to 2017/2018 seasons, while the outer short dashed blue lines show the three week buffer about this historic window. The validation ILI values are shown in black. For Puerto Rico in 2013 and the Virgin Islands in 2014, the `NA` occurs within the buffered historic window and leads the seasonal targets in those state-seasons to be un-scorable. In the District of Columbia in 2015, on the other hand, the `NA` occurs outside the buffered historic window and so seasonal targets for that state-season are scorable.

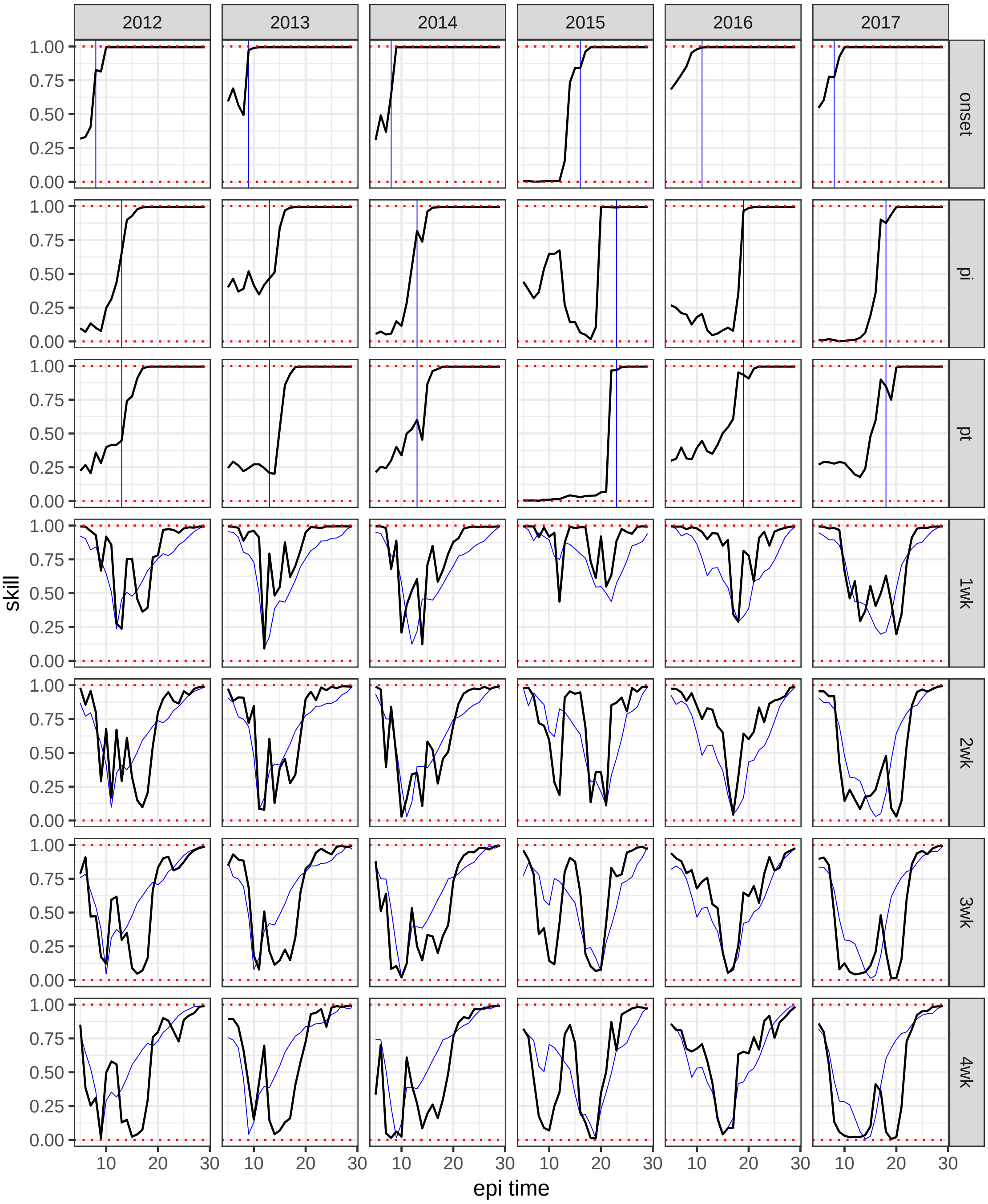

Supplementary Figure 9: Dante's forecast skill by prediction time for each target at the national level (black line). The validation onset (peak timing) for each season is shown in the first (second/third) row as a vertical blue line. Shifted national wILI data for each season multiplied by -1 is shown as the thin blue line overlaid on the short-term target subplots (scaled so as to have the same range as skill in each subplot) For $n-$week ahead forecasts $-$wILI is shifted back n weeks such that $-$wILI for the week being predicted is overlaid on the week in which the prediction was made. It is evident that short-term target skill is inversely related to wILI.

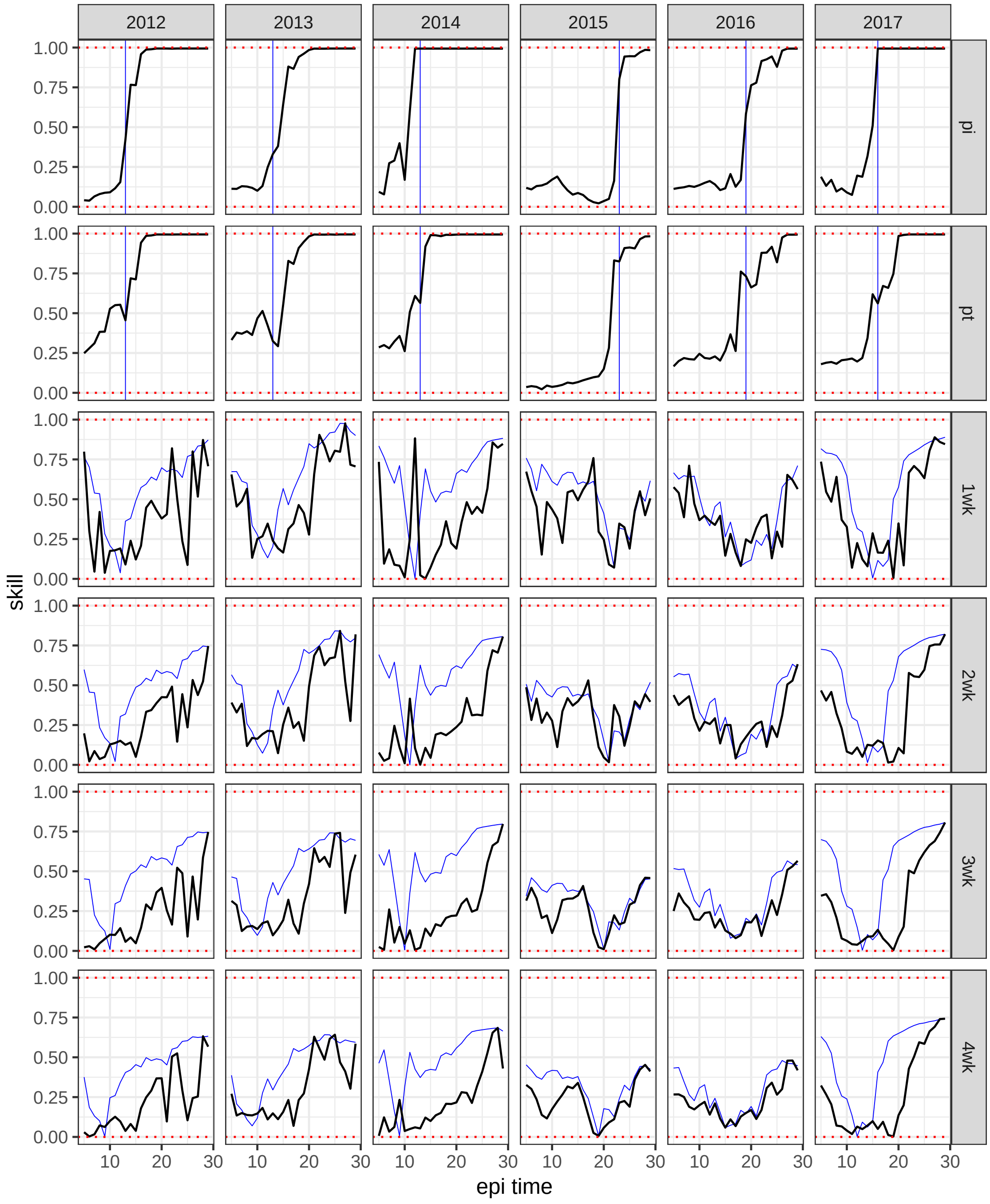

Supplementary Figure 10: Dante's forecast skill by prediction time for each target in Alabama (black line). The validation onset (peak timing) for each season is shown in the first (second/third) row as a vertical blue line. Shifted ILI data for each season multiplied by -1 is shown as the thin blue line overlaid on the short-term target subplots (scaled so as to have the same range as skill in each subplot) For $n-$week ahead forecasts $-$ILI is shifted back n weeks such that $-$ILI for the week being predicted is overlaid on the week in which the prediction was made. Short-term target skill tends to decrease as ILI increases.

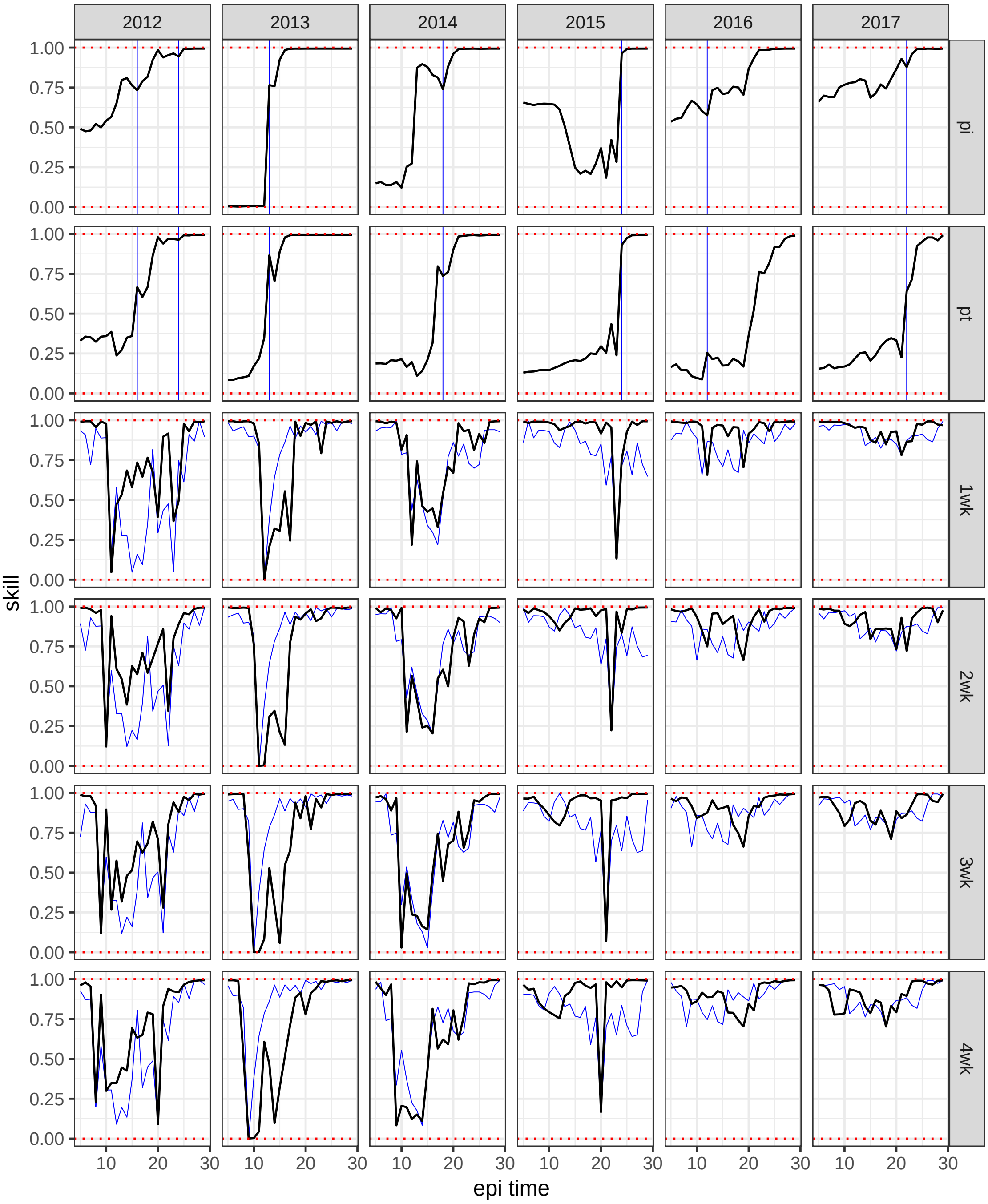

Supplementary Figure 11: Dante's forecast skill by prediction time for each target in Montana (black line). The validation onset (peak timing) for each season is shown in the first (second/third) row as a vertical blue line or lines. Shifted ILI data for each season multiplied by -1 is shown as the thin blue line overlaid on the short-term target subplots (scaled so as to have the same range as skill in each subplot) For $n-$week ahead forecasts $-$ILI is shifted back n weeks such that $-$ILI for the week being predicted is overlaid on the week in which the prediction was made.

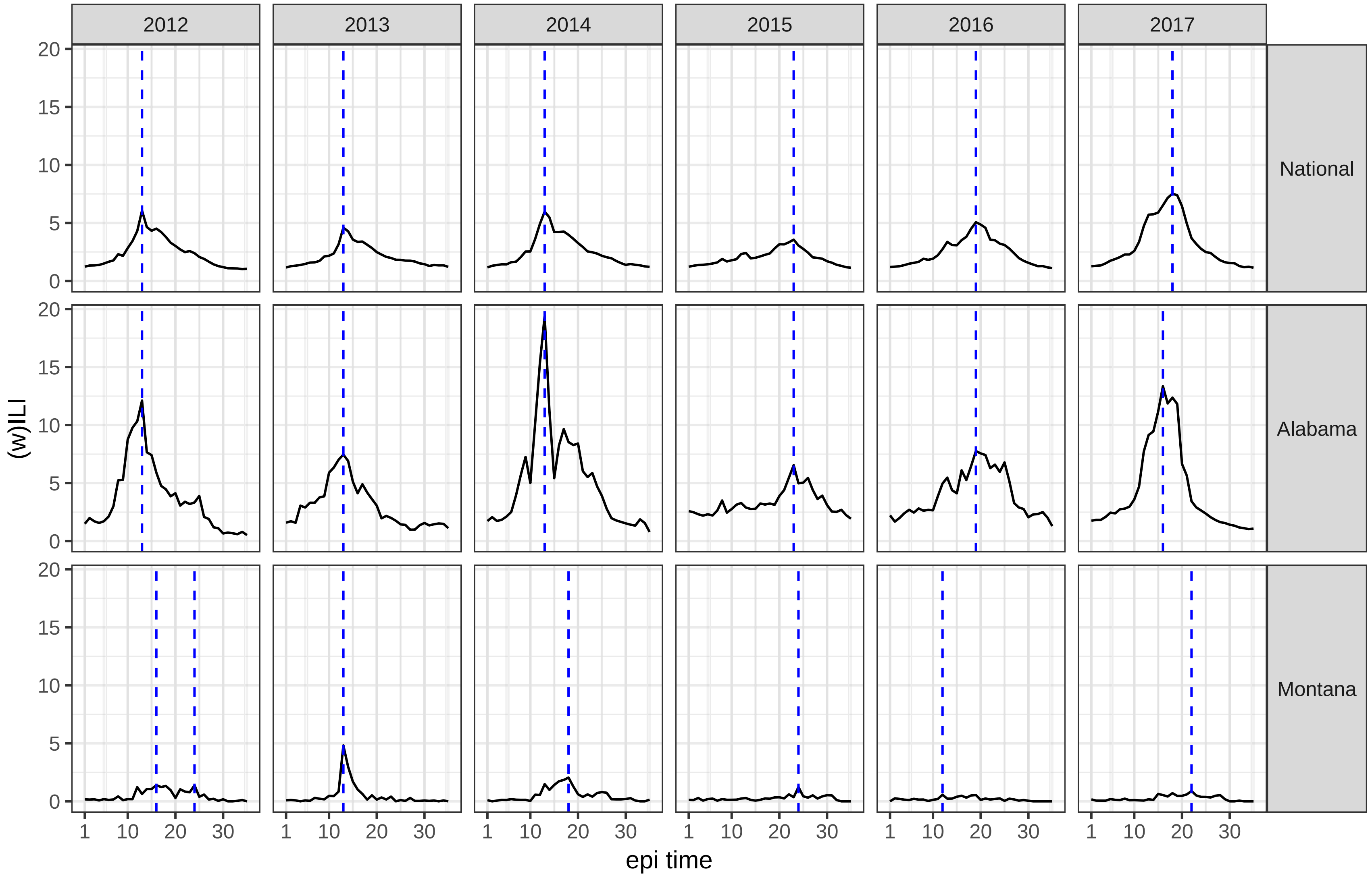

Supplementary Figure 12: National wILI data and state ILI data for the example skill plots discussed. This figure is intended as a reference for magnitude of (w)ILI data because the -(w)ILI data in the skill plots has been scaled to lie in the same range as skill. Note that Alabama has exceptionally high ILI relative to the national average, while Montana has particularly low ILI relative to the national average. The vertical dashed blue line marks the peak week(s) for each region-season combination.

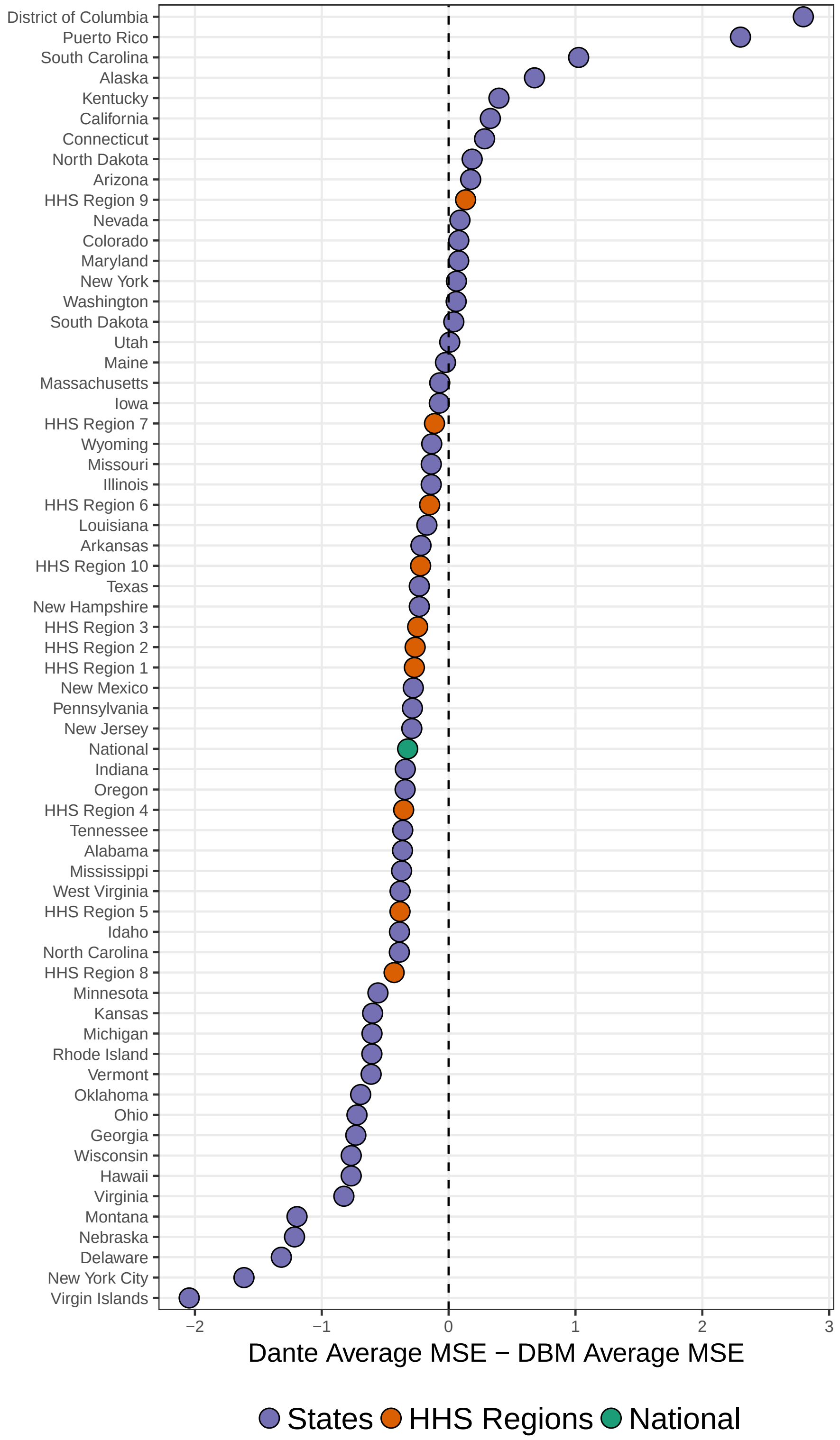

Supplementary Figure 13: Difference in mean squared error (MSE) between Dante and DBM, for all states, regions, and nationally. Dante had lower MSE for the majority of geographic regions.

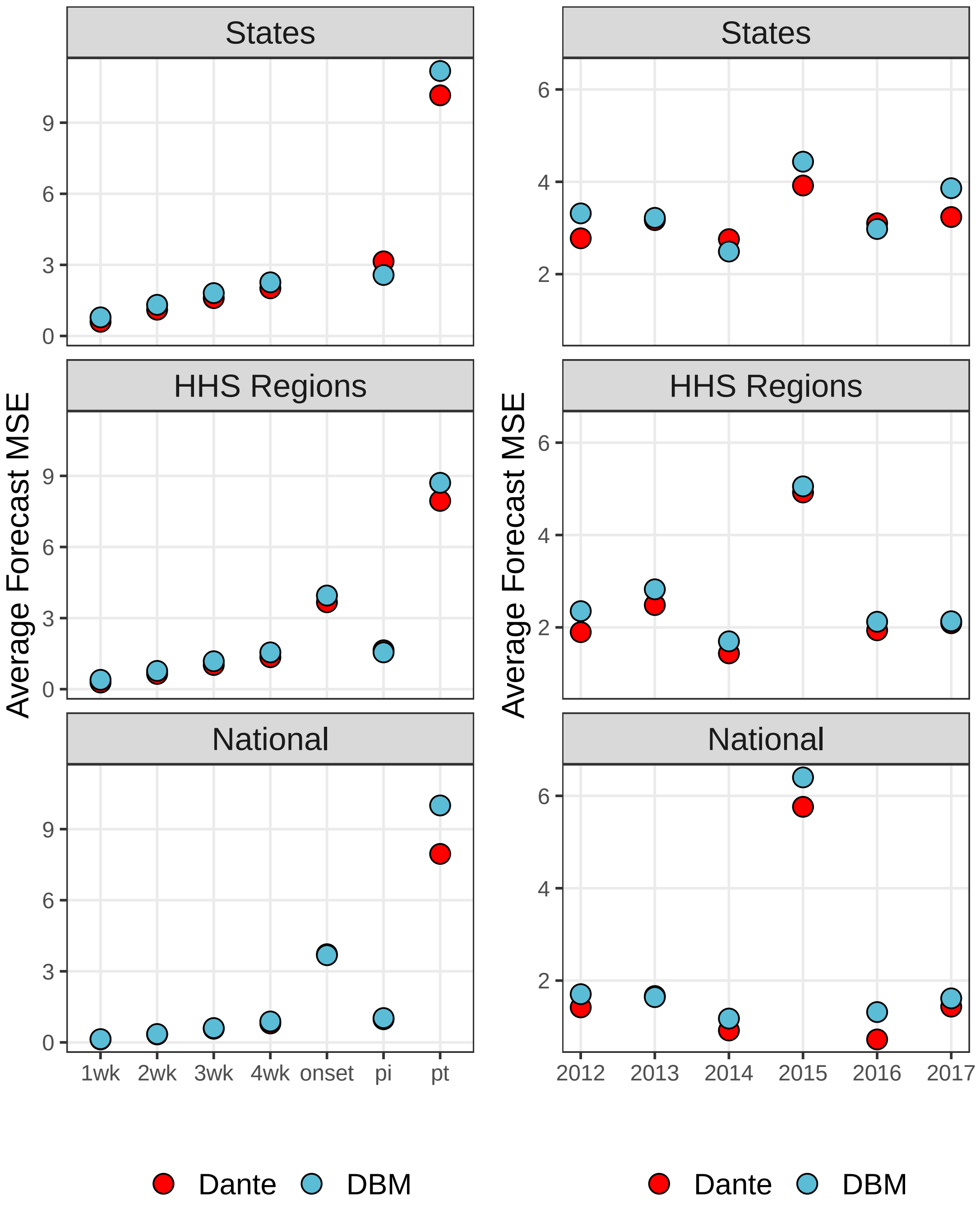

Supplementary Figure 14: (left) Average mean squared error (MSE) by scales and targets. PI and PT stand for peak intensity and peak timing, respectively. Dante outperformed DBM for all scales and targets, except for onset nationally and for peak intensity (PI) regionally and by state. (right) Average forecast skill by scales and flu seasons. Dante outperformed DBM for the majority of scales and flu seasons, except for 2013 nationally and for 2014 and 2016 by state.
